# New *Dielis* species and structural dichotomy of the mitochondrial *cox2* gene in Scoliidae wasps

**DOI:** 10.1038/s41598-023-27806-x

**Published:** 2023-02-02

**Authors:** Przemyslaw Szafranski

**Affiliations:** Department of Molecular and Human Genetics, Baylor College of Medicine, One Baylor Plaza, Houston, TX 77030 USA

**Keywords:** Evolution, Genetics, Molecular biology, Zoology

## Abstract

Some mitochondrial protein-coding genes of protists and land plants have split over the course of evolution into complementary genes whose products can form heteromeric complexes that likely substitute for the undivided proteins. One of these genes, *cox2*, has also been found to have split in animals, specifically in Scoliidae wasps (Hymenoptera: Apocrita) of the genus *Dielis* (Campsomerini), while maintaining the conventional structure in related *Scolia* (Scoliini). Here, a hitherto unrecognized Nearctic species of *Dielis*, *D. tejensis*, is described based on its phenotype and mtDNA. The mitogenome of *D. tejensis* sp. nov. differs from that of the sympatric sibling species *Dielis plumipes fossulana* by the reduced size of the *cox2*-dividing insert, which, however, still constitutes the fifth part of the mtDNA; an enlarged *nad2-trnW* intergenic region; the presence of two *trnK*^*ttt*^ paralogues; and other features. Both species of *Dielis* have a unique insertion of a threonine in COXIIA, predicted to be involved in COXIIA-COXIIB docking, and substitutions of two hydrophobic residues with redox-active cysteines around the Cu_A_ centre in COXIIB. Importantly, the analysis of mtDNA from another Campsomerini genus, *Megacampsomeris*, shows that its *cox2* gene is also split. The presented data highlight evolutionary processes taking place in hymenopteran mitogenomes that do not fall within the mainstream of animal mitochondrion evolution.

## Introduction

Proteins encoded by genes resulting from ancient gene fissions can form heteromeric complexes that are thought to function like the ancestral undivided proteins (e.g., Ref.^1^). The proof-of-principle for the ability of such quasi-native macromolecular structures to retain the activities attributed to undivided proteins has been demonstrated by numerous complementation assays (reviewed in Ref.^2^). Evolutionary gene fragmentations into apparently complementary genes have been reported from all major branches of life, viruses and mitochondria; in the case of mitochondria most examples are in protists, but also liverworts and some flowering plants (e.g., Ref.^3,4^). In total, orthologues of ten mitochondrial protein-coding genes (PCGs) have been found to be split in some organisms into two (*atp8*, *ccmF*, *cox1*, *cox2*, *nad1*, *nad2*, *nad5*, *rpl2*, *rps3*, and *sdhB*) or three (*ccmF*) genes (compiled in Ref.^5–7^). The only known evolutionary fission of an animal mitochondrial gene and its protein was described for *cox2* in digger wasps (Hymenoptera: Apocrita: Scoliidae) of the genus *Dielis* (tribe Campsomerini)^8^.

Scoliidae is a cosmopolitan but predominantly subtropical and tropical family of large solitary aculeates with approximately 560 extant species and 220 subspecies^9^, whose larvae are ectoparasitoids mostly of soil-inhabiting Scarabaeoidea beetle grubs. With two exceptions, all extant species of Scoliidae are almost equally divided between tribes Scoliini and Campsomerini within the subfamily Scoliinae.

The *cox2* gene encodes the essential subunit II of cytochrome c oxidase (COX, complex IV) of the mitochondrial respiratory chain (reviewed in, e.g., Ref.^10^). Canonical COXII features a short N-terminal intermembrane domain, two trans-inner-membrane α-helices and an intermembrane-space-exposed C-terminal domain, containing a dinuclear copper centre, Cu_A_, that directs electron transfer from reduced cytochrome c to dioxygen bound between haem a_3_ and Cu_B_ in COXI.

*Cox2* is also interrupted by 0.3–4.5 kb insertions in male-transmitted mitogenomes of venerid bivalve molluscs with doubly uniparental inheritance of mtDNA^7,11,12^. However, studies of the marine clam *Scrobicularia plana*, showed that its *cox2* gene is expressed as an enlarged rather than split protein^12^. Of note, with the exception of those of certain annelids^13,14^, the mitogenomes of bilaterian animals are devoid of intron-containing genes. Likewise, there have been no reports of intein coding by any mtDNA. In addition to *Dielis*, split COXII proteins are known in apicomplexans, dinoflagellates (Alveolata)^15–18^ and chlorophycean algae (Chlorophyta)^19–22^, in which one or both of the *cox2*-derived genes relocated to the nuclear genome. In ciliates (Alveolata), bicosoecids and brown algae (Heteroconta), *cox2* is split by insertions that are thought to be translated in-frame with flanking *cox2* fragments into a single enlarged COXII polypeptide (e.g., Ref.^23–25^).

In the course of research aimed at better defining the group of Scoliidae carrying a bipartite *cox2* locus, a hitherto unknown Nearctic species of *Dielis* was found and is described here. In its current concept, the genus *Dielis* Saussure and Sichel (formerly a subgenus of *Campsomeris* Guérin) consists of nine species and three subspecies, with one exception, of southern Nearctic and Neotropical distribution^9,26^. The unchanging core of this group includes *D. plumipes* (Drury) (the type species of *Dielis*), *D. plumipes confluenta* (Say), *D. plumipes fossulana* (Fabricius), *D. trifasciata* (Fabricius), *D. trifasciata nassauensis* (Bradley), *D. tolteca* (Saussure), *D. bahamensis* (Bradley) and *D. dorsata* (Fabricius).

This report also draws attention to the loss of *cox2* gene integrity due to large intragenic insertion in yet another genus of Campsomerini, *Megacampsomeris* Betrem. Finally, it addresses, equally atypical in bilaterian animals, frequent enlargement of intergenic regions in the mitogenomes of Hymenoptera.

## Results

### New species account

*Taxonomy*: Arthropoda; Hexapoda; Insecta; Pterygota; Neoptera; Endopterygota; Hymenoptera; Apocrita; Aculeata; Scolioidea; Scoliidae; Scoliinae, sensu Raznitsyn^27^; Campsomerini; *Dielis* Saussure & Sichel, 1864^28^.

*Dielis tejensis* sp. nov.

urn:lsid:zoobank.org:act:7E4E00DB-180B-427A-886A-13FB4DF53F6D.

**Figure 1:**
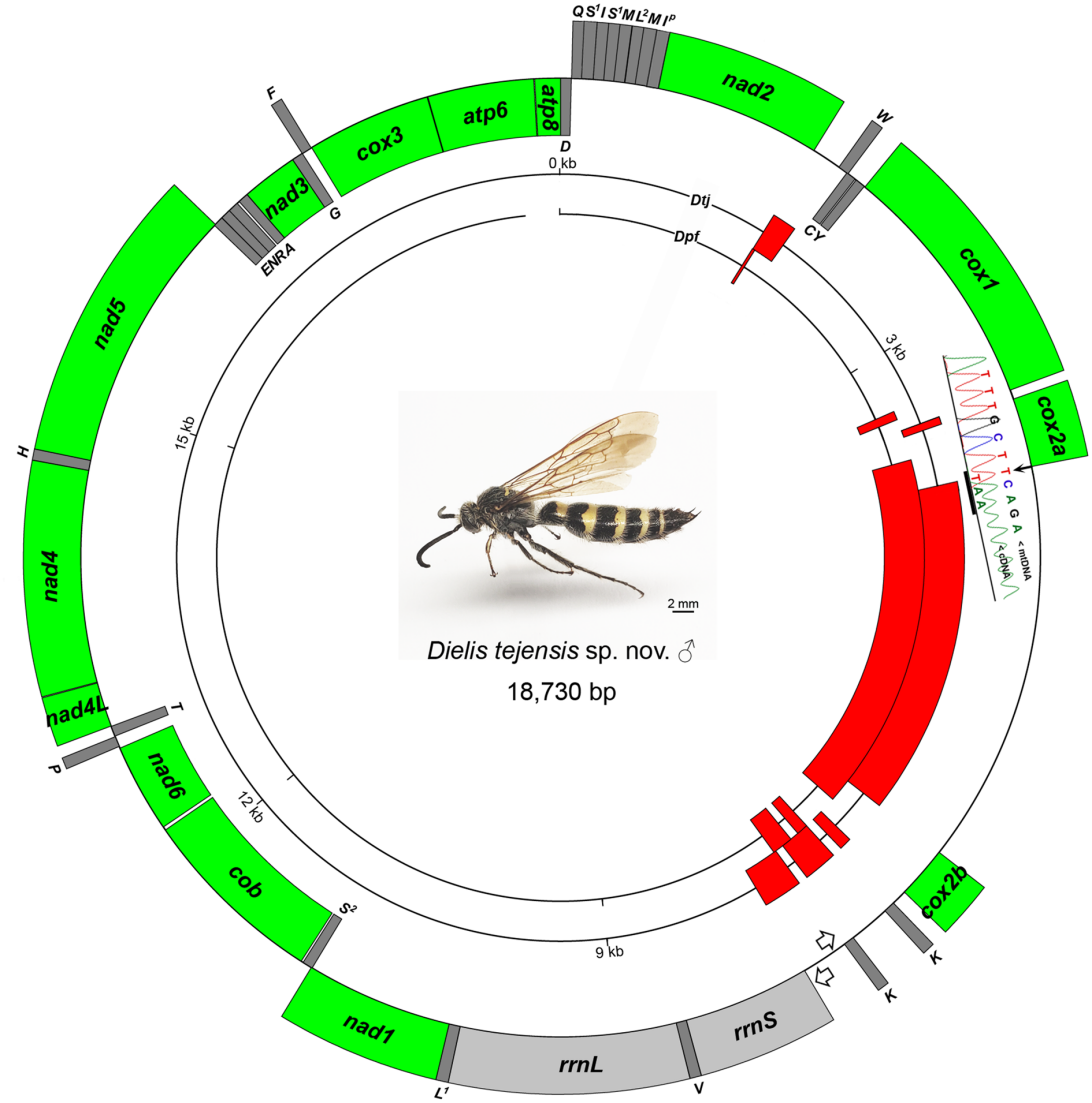
*D. tejensis* sp. nov. and its mitochondrial genome. Open arrows indicate the location of putative promoters and directions of polycistronic transcription; capital letters represent *trn* gene symbols (*I*^*p*^, putative *trnI* pseudogene). The electropherogram shows the cDNA sequence around the *cox2a* 3′-end with termination codon T(AA) completed by polyadenylation. Concentric circles represent the mitogenomes of *D. tejensis* sp. nov. (*Dtj*) and *D. p. fossulana* (*Dpf*) (the last one is arbitrarily interrupted), with red marked location of intergenic regions larger than 25 bp, including the *cox2*-splitting insert.

*Habitus*: Fig. 1, Supplementary Fig. S1.

*Type material*: Holotype male, ENTO X1194700: USA, Texas, [Williamson Co.], Taylor, 20/IV/1963, J. E. Hafernik leg., (TAMU). Paratypes are listed in Supplementary Data S1.

*Etymology*: The latinized specific epithet refers to a historical name of the state of Texas, Tejas, where this species has been recorded.

**Figure 2:**
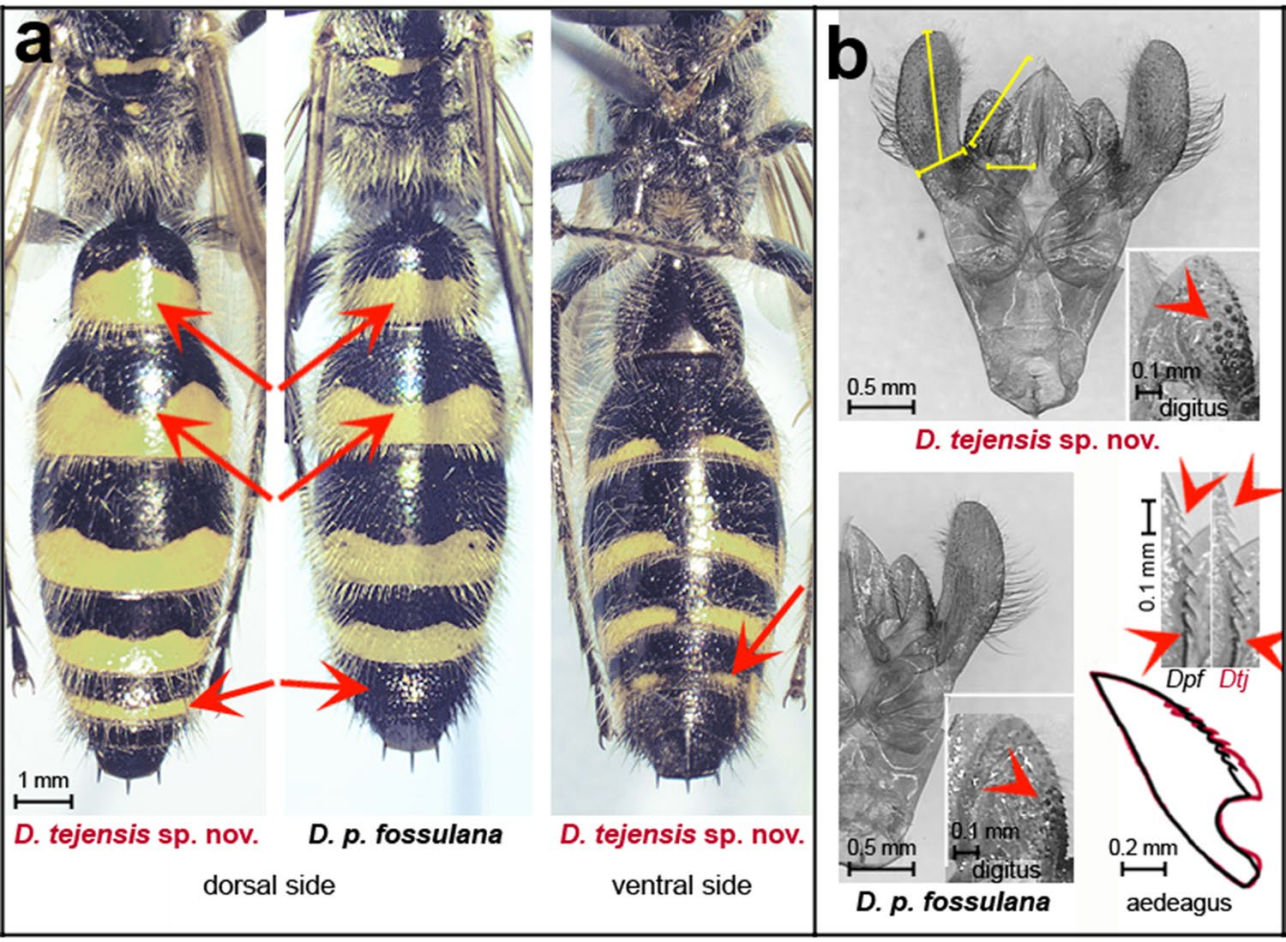
Selected phenotypic characters distinguishing *D. tejensis* sp. nov. (*Dtj*) from the most closely related species *D. p. fossulana* (*Dpf*). (**a**) Metasoma. (**b**) Dorsal aspect of terminalia (aedeagus blades are spread perpendicular to their natural position); digitus volsellaris in ventral view; serrated margin of the aedeagus; and alignment of the shapes of the aedeagus blades. Yellow lines indicate how the length and width of the paramere and aedeagus blade were measured. Red arrows indicate differences in the colour pattern on the metasoma, arrowheads point at sensory cones on the digitus, and the 1st and last teeth on the edge of the aedeagus.

*Diagnosis*: Males (female unknown) most closely resemble the sympatric species *D. p. fossulana*, from which they differ by having slightly more slender basal segments of the metasoma, a slightly wider paramere and aedeagus blades, less developed 1st and last teeth on the edge of the aedeagus, larger sensory cones occupying a larger area on the digitus volsellaris (Fig. 2) (all these morphological differences become more apparent when comparing longer series of specimens), a yellow transverse band on the pronotum (Supplementary Fig. S2) and an apical band on the 5th metasomal segment (Fig. 2). The mitogenome is distinguished by fixed nucleotide substitutions, often translating to amino acid substitutions, the size of some intergenic regions, *trnK* gene duplication, and other features (Fig. 1, Supplementary Fig. S3, Supplementary Table S1). Verification of phenotype-based identifications of this species can be done by PCR amplification and sequencing of, e.g., a 0.3 kb *cox1* fragment containing 16 *D. tejensis* sp. nov.-specific nucleotide positions, using primers co1F1 and co1R2 (Supplementary Data S2).

*Description of the phenotype*: Morphology (n = 10)—Body length 17.8 ± 2.1 mm (SD), fore-wing length 14.3 ± 1.5 mm (SD). Head: frontal fissure merely indicated, frons largely impunctate below the anterior ocellus but contains the central pit, laterally features two weakly defined punctate glabrous tubercles with bristles arising from pits, hypostomal carina without submandibular triangle. Antenna: scape and pedicel punctate and covered with bristles. Mesosoma: propodeum in lateral view not rounded immediately behind the scutellum, making instead an obtuse angle with the posterior vertical part, lateral carina discontinuous beyond the spiracle. Wings: forewing with two submarginal cells, the 2nd of which receives two recurrent veins. Legs: spurs of the hind tibia unequal in length, inner spur spatulate. Metasoma: ratio of the median length of the 1st tergite (in vertical projection without stalk) to its maximal width 0.88 ± 0.06 (SD). Terminalia: volsella transversely divided, the length-to-width ratios for paramere and aedeagus blade 2.6 and 2.4, respectively (n = 2) (Fig. 2). Colouration—Integument mostly black with yellow pattern that includes eye inner margin, base of mandible, stripes/spots on the pronotum, scutellum and metanotum, apical bands on the 1st–5th metasomal tergites and 2nd–5th sternites, broadly interrupted on the sternites (Fig. 2), basal spot on the tegula, dorsal line on the protibia, extending to both adjacent leg segments, a spot on the antero-lateral side close to the apex of mezo- and metafemur present in half of the examined specimens (Supplementary Fig. S2). Antenna black-brown; protarsi brown, spurs of the hind tibia white; vestiture yellowish-white, yellowish-white and black on the 5th metasomal segment, and black on the 6th and 7th segments (for a more detailed description, see Supplementary Data S3).

**Figure 3:**
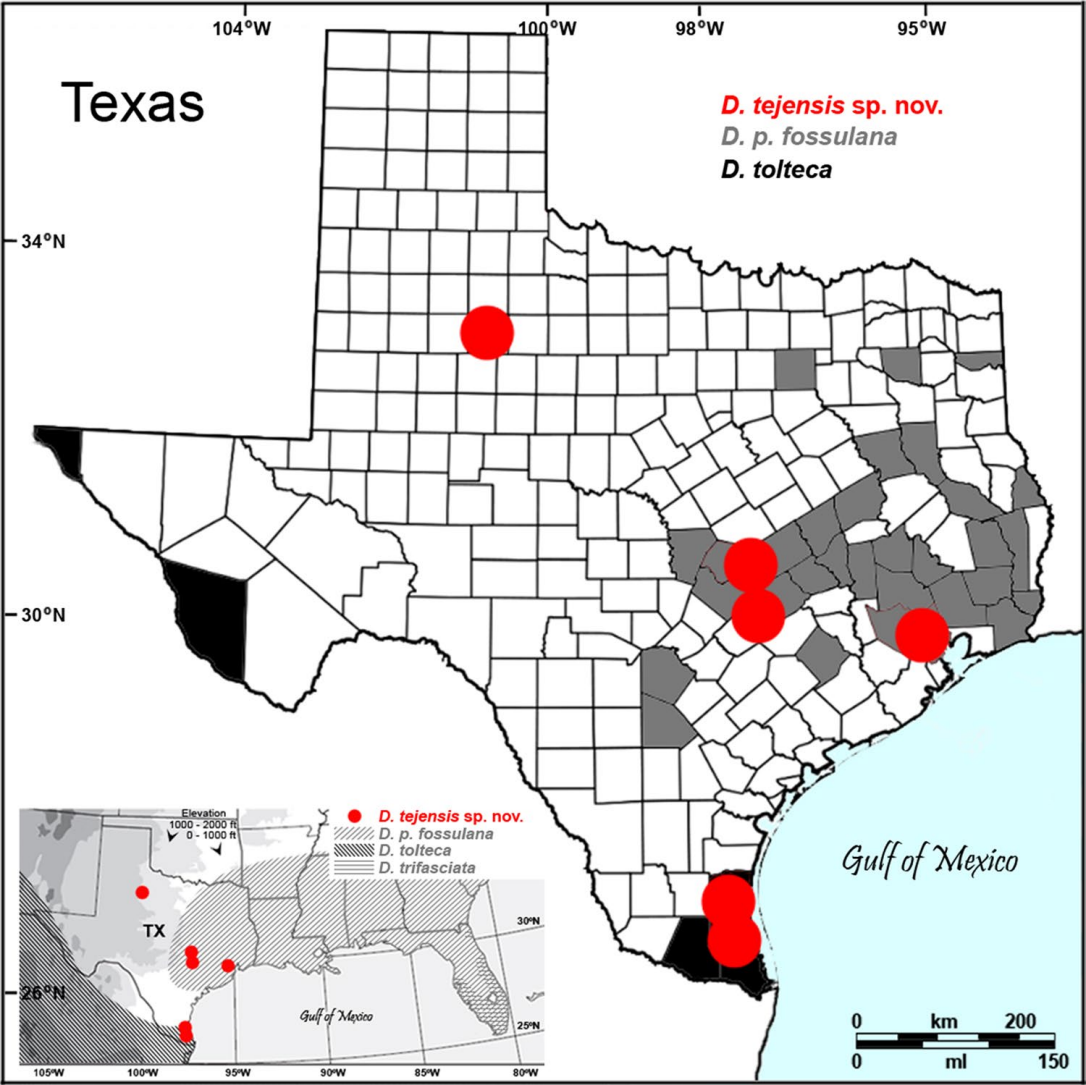
Geographic distribution of *D. tejensis* sp. nov. and similar species of *Dielis*. Contemporary and historical (published^30^, TAMU and FSCA) records are plotted on contour maps obtained from Wikimedia Commons and modified.

*Distribution*: Southern Nearctic: eastern Texas, from the Low Rolling Planes, southeast of the Caprock Escarpment, to the Gulf Coastal Plains (Fig. 3).

*Habitat*: Austroriparian life zone, mostly in humid, subtropical climate, at altitudes up to 600 masl. Flower records include herbaceous and shrubby Asteraceae *Eupatorium serotinum* and *Baccharis halimifolia*, respectively, at the edges of mixed deciduous forests, mesquite woods, in thickets and post oak savannah. The host of this wasp is unknown. Larvae of *D. p. confluenta* and *D. trifasciata* parasitize grubs of the scarabs *Cotalpa lanigera* (Rutelinae)^29^ and *Phyllophaga plaei* (Melolonthinae)^30^, respectively.

### The unorthodox mitochondrial genome of *D. tejensis* sp. nov

*General characteristics of the mtDNA* (Fig. 1, Supplementary Table S1): The mitogenome of *D. tejensis* sp. nov. is 18,730 bp in size, which is larger than the average bilaterian animal mitogenome (16.5 kb). This size increase is mainly due to the presence of the 2.5 kb insertion splitting the *cox2* gene. The *D. tejensis* sp. nov. mtDNA is composed of 79% adenine (A) and thymine (T) bases and contains all genes found in typical bilaterian mtDNA. Relative synonymous codon usage scores indicate more frequent use of A and T in *D. tejensis* sp. nov. compared to *Drosophila* (Supplementary Table S2). PCGs are predicted to start with the ATD codon. Nine of them apparently end with the existing stop codon TAA, and two end with TGA. Only *cox2a* (Fig. 1) and *nad4* genes end with the abbreviated stop codon T, which, as shown by 3’ RACE, is completed by polyadenylation. Thus, *D. tejensis* sp. nov. initiation and termination codon usage follows the “codon conventionalization” trend seen in other fast-evolving mtDNAs of apocritan Hymenoptera^31^. Three predicted tRNAs have anticodons (Supplementary Table S1) that differ in wobble position from those in the majority of insects. They include tRNA-G (acc instead of more common ucc), tRNA-K (uuu instead of cuu) and tRNA-S1 (acu instead of gcu).

The control region (CR) is 0.6–0.8 kb in size (Supplementary Fig. S4). Its AT content is similar to the average AT content of the mitogenome. Most of the CR consists of a tandem duplication of a 0.3 kb segment containing *trnK*^*ttt*^. The remaining part, abutting the *rrnS* gene, comprises AT-rich short tandem repeats. Two putative transcriptional promoters (P_H_ and P_L_) and several origin of replication (O_R_)-like palindromic structures were identified within this region on the basis of their similarity to functionally verified human mitochondrial promoters and O_R_s^32,33^. The promoter location was independently confirmed using the NNPP program.

The mitogenomes of *D. tejensis* sp. nov. and *D. p. fossulana* (GenBank_KT740996) share BLAST-determined regions of mutual sequence homology divergent for those 1 kb and larger at 6% to 13% of nucleotide positions (Supplementary Fig. S3). The corrected genetic distance between the barcode genes *cox1* of *D. tejensis* sp. nov. and *D. p. fossulana*, calculated using the GTR substitution model, is 6.6% whereas *cox1* intraspecific variability for each of the taxa at the same location (Texas) is 0.2%.

*Gene rearrangements*: In *D. tejensis* sp. nov. as well as in *D. p. fossulana*, *trnF*^*gaa*^, *trnL2*^*taa*^ and *trnS1*^*a/tct*^ individually relocated, and in the last case, they also underwent duplication with an anticodon change, while *trnQ*^*ttg*^ inverted and swapped position with *trnI*^*gat*^ (Supplementary Fig. S5, Supplementary Table S1). Rearrangements of tRNA-coding genes are rather common in apocritan Hymenoptera (e.g., Ref.^34–36^). In contrast, PCG inversions or translocations, especially those involving gene clusters, have been reported much less frequently, primarily in Chalcidoidea (e.g., Ref.^37,38^). Similar to *D. p. fossulana*, the mitogenome of *D. tejensis* sp. nov. has a 7.1-kb-long segmental inversion between the *trnD*^*gtc*^ gene and the CR that includes, in addition to several *trn* genes/pseudogenes, PCGs *nad2*, *cox1*, *cox2a* and *cox2b*. Because of this inversion, there is almost no weight difference between the L and H strands of the mtDNA (GT content 49.97% versus 50.03%, respectively). A similar inversion has been found in *Scolia* (Scoliini)^8^ and may be common to Scoliinae. It could have occurred through replication-based nonallelic homologous recombination between two ancestral clusters of tRNA genes, *trnI* + *trnQ* + *trnM* and *trnK* + *trnD*, the former of which is already known as a rearrangement hotspot (e.g., Ref.^34^), or through generation of a minicircle and its integration back into the mitogenome^39^.

*Duplicated tRNA genes*: Regions at the edges of the abovementioned segmental inversion harbour supernumerary *trnM*^*cat*^, *trnS1*^*a/tct*^, *trnI*^*g/aat*^ (CR-*nad2* region) and *trnK*^*ttt*^ (*cox2b*-CR region) genes/pseudogenes. High tRNAscan covariance scores (cov) of *trnM*^*cat*^ and *trnK*^*ttt*^ paralogues (19.5, 26.3 and 29.9, 44.0, respectively) suggest that the tRNAs encoded by the *trn* duplicates are functional. Apart from *Dielis*, *trnM*^*cat*^ is apparently duplicated in the syntenic location in *Scolia* (GenBank_KT276222), Eumeninae (Vespidae) (e.g., Ref.^40^), some species of *Apis* (e.g., GenBank_KY348372) and *Bombus* (e.g., GenBank_MK570129) (Apidae), and some Chalcididae (e.g., GenBank_MG923487), and it is triplicated in Ibaliidae (Cynipoidea)^41^. In *D. tejensis* sp. nov., the two *trnM*^*cat*^ genes are 63% identical (E < 1.7e−3, Supplementary Fig. S6a). They are separated by *trnL2*^*taa*^, which based on pairwise sequence alignments (Supplementary Fig. S6b) might have originated through the conversion of a copy of one of *trnM*^*cat*^s (mtDNA coordinates: 331–398; identical to *trnL2*^*taa*^ in 52%, E < 8.2e−2). The *trnL2*^*taa*^ gene is also located in the syntenic region upstream of *nad2* in *Scolia* (GenBank_KT276222), Chrysididae (e.g., Ref.^42^), the majority of Ichneumonidae (e.g., Ref.^36^) and Cynipoidea (e.g., Ref.^41^).

The *trnS1*^*a/tct*^ genes are in 68% identical (E < 1.5e−2), but only *trnS1*^*tct*^ was detected by both ARWEN and MITOS (Supplementary Fig. S6c). Of note, there are three *trnS2*^*a/tga*^ pseudogenes, located within the *rrnL* and *nad1* genes, with no sequence similarity to other *trn* genes. As with those *trnS2*s, *trnI*^*g/aat*^s are not paralogous. Inferring from the low cov value of 2.8, *trnI*^*aat*^ is likely a pseudogene.

The two *trnK*^*ttt*^ genes, located at the other end of the inverted region, are 85% identical (E < 8.2e−09) (Supplementary Fig. S6d). In hymenopteran mitogenomes, *trnK* duplication is otherwise known only in one species of unclassified Scoliidae (GenBank_MH748661).

**Figure 4:**
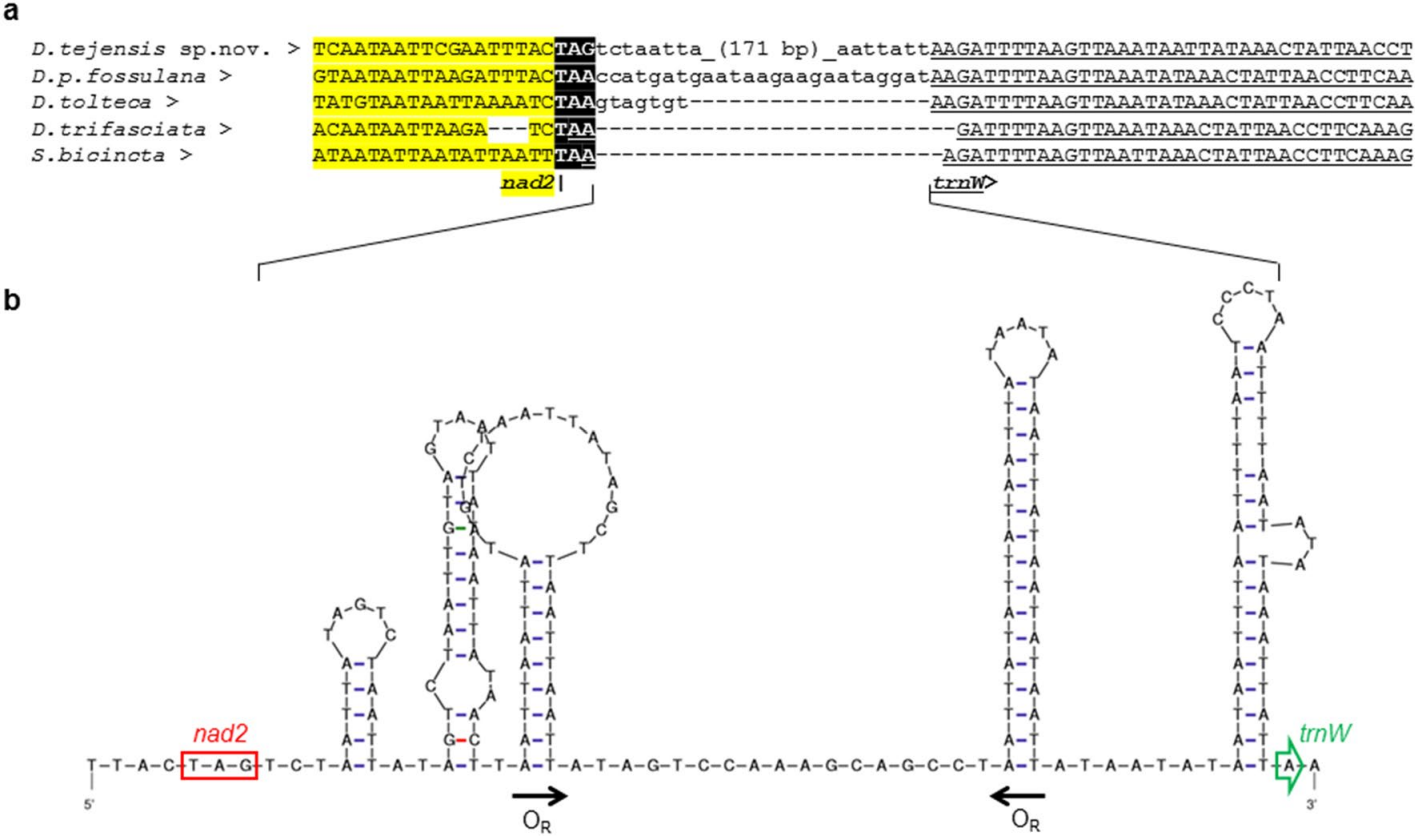
Intergenic *nad2-trnW* region in Scoliidae mitogenomes. (**a**) Multiple sequence alignment of the *nad2-trnW* interval (L strand; *trnW* is underlined). (**b**) Predicted secondary structure of the *D. tejensis* sp. nov. *nad2-trnW* region in the single-strand conformation (mfold dG = − 19.06 kcal/mol). O_R_, putative origin of replication.

*Enlarged intergenic regions*: Unlike those in the majority of bilaterian mitogenomes, some intergenic regions in the mtDNA of *D. tejensis* sp. nov. are enlarged. For instance, the region between the *nad2* and *trnW* genes is 186 bp in size (Fig. 4). The syntenic region in *D. p. fossulana* is 25 bp long, comprises only 7 bp in *D. tolteca* and is absent in *D. trifasciata* and *Scolia bicincta* (Figs. 1, 4a). The mfold analysis of the *nad2-trnW* interval showed that it could adopt palindromic structures resembling two oppositely oriented alternative O_R_s (Fig. 4b). Each of them has a moderately T-enriched loop that may initiate RNA primer synthesis for DNA replication and is adjacent to a GC-rich sequence resembling the motif associated with the RNA–DNA transition/cleavage site^32^.

Another intergenic region, 58 bp long, lies between *cox1* and *cox2a* (Supplementary Fig. S7). Part of it may have arisen as a result of *trnL2* pseudogenization which left behind palindromic structures that possibly play a role in generating the 3′- and 5′-ends of *cox1* and *cox2a* mRNAs, respectively (Supplementary Fig. S7). The *cox1-cox2a* interval is also enlarged in *D. p. fossulana* (Supplementary Table S1).

There is also a 95-bp noncoding region located between *cox2b* and *trnK*^*ttt*^ that may be part of the neighbouring CR. However, the largest intergenic interval consists of or is included in the *cox2*-splitting insert described below.

**Figure 5:**
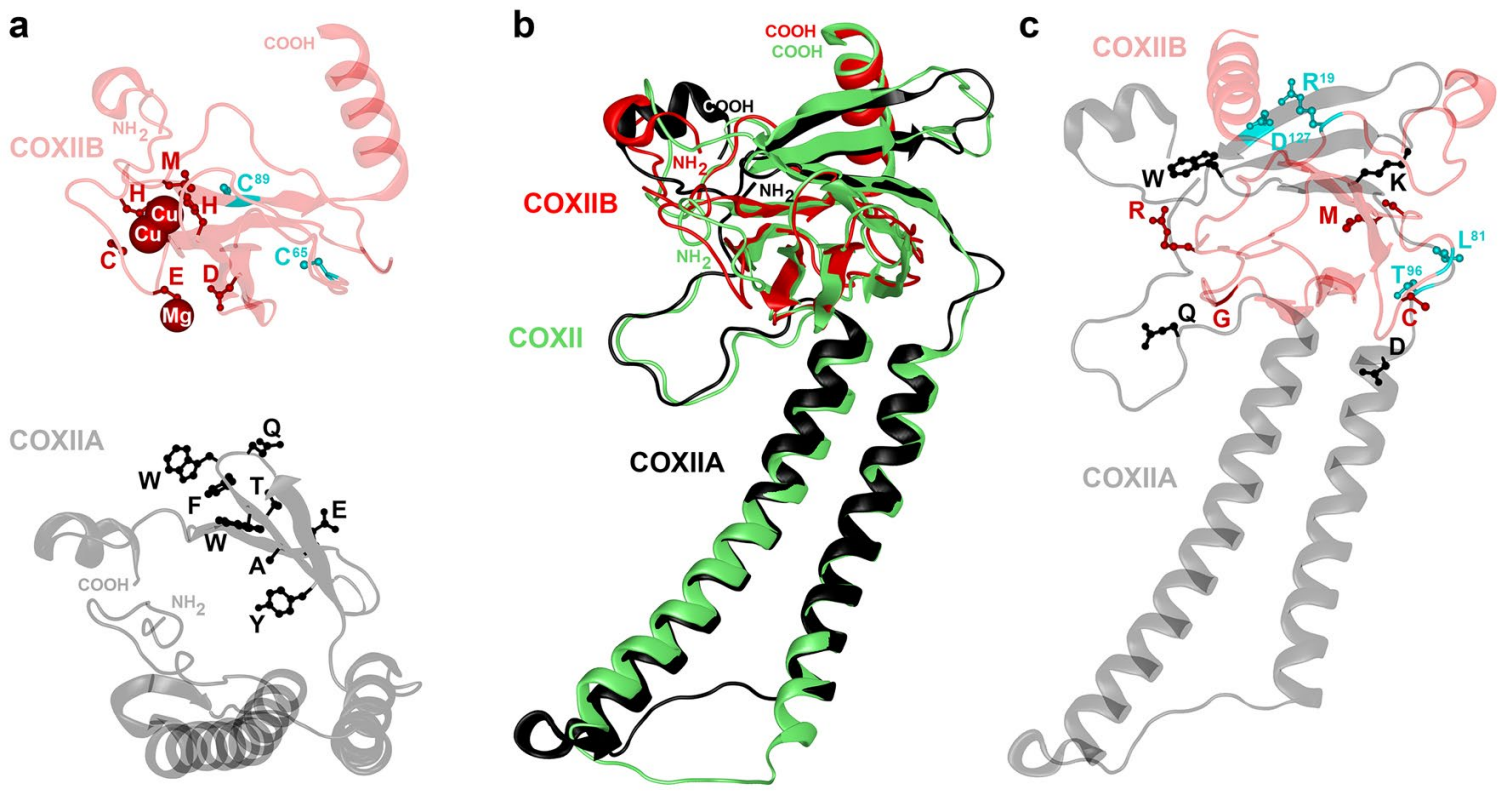
Ribbon diagrams of the predicted three-dimensional structures of COXIIA, COXIIB and their complex in *D. tejensis* sp. nov. Sequences of the analysed proteins are shown in Supplementary Fig. S8. The folding of split and canonical COXIIs was modelled on the crystal structures of bacterial (PDB accession numbers: 3HB3, 1M56, and 4TXV), yeast (6T15) and bovine (1OCZ) cytochrome c oxidases. (**a**) Tertiary structures of COXIIA and COXIIB. Amino acid residues of the COXIIA “haem patch” are shown in black; COXIIB residues chelating magnesium and mixed-valence copper ions in the Cu_A_ centre are shown in red; unique to *Dielis* Cys residues flanking the Cu_A_ centre are in blue. COXIIA is pictured in a plane view approximately perpendicular to its projections in the next panels. (**b**) The alignment of the tertiary structures of the *D. tejensis* sp. nov. COXIIA (black), COXIIB (red) and undivided COXII of *S. bicincta* (green). (**c**) The most stable COXIIA-COXIIB docking decoy (-24.3 kcal/mol). Selected evolutionarily conserved residues involved in docking interactions (Supplementary Fig. S8) are shown in black (COXIIA) and red (COXIIB), supernumerary Thr unique to *Dielis*, its interactor Leu, and a pair of residues Asp and Arg, predicted to form a salt bridge between COXIIA and COXIIB, are marked in blue.

*Split* cox2 *gene and its protein*: The most distinguishing feature of *D. tejensis* sp. nov. mitogenome is the bipartite structure of the *cox2* locus, which consists of complementary *cox2a* and *cox2b* genes. No consensus splicing sites or structures resembling Group I or II introns or sequence similarity to inteins was found within and around the insert that splits *cox2*. End-point RT-PCR using primers flanking this insert also did not reveal evidence for *cox2* mRNA splicing. Likewise, no heteroplasmy was detected by PCR using mtDNA and primers flanking the insert. 3′ RACE showed that the *cox2a* termination codon T(AA) is completed posttranscriptionally (Fig. 1). It maps to the base of a palindrome whose localization correlates with the abrupt loss of sequence similarity to conventional *cox2* genes. Thus, the *D. tejensis* sp. nov. *cox2* locus is likely expressed as its orthologue in *D. p. fossulana*, where *cox2a* and *cox2b* are separate genes translated into two separate polypeptides COXIIA and COXIIB^8^. The predicted tertiary structures of *D. tejensis* sp. nov. COXIIA and COXIIB are shown in Fig. 5a. The “haem-patch” region, containing the Trp residue involved in electron transfer from cytochrome c, is included in COXIIA, whereas the Cu_A_ centre is included in COXIIB. The structure of the *D. tejensis* sp. nov. COXIIA-COXIIB heterodimer (MW 29,405.68 g/mol), modelled on canonical complex IV, resembles the folding of an undivided COXII protein in *S. bicincta* (Fig. 5b). The amino acid residues interacting at the docking interface in the most stable COXIIA-COXIIB docking decoy (65 amino acid contacts) (Fig. 5c, Supplementary Fig. S8, Supplementary Table S3) were clustered in subregions corresponding to those predicted previously for the COXIIA/COXIIB interface in the alga *Polytomella* sp.^43^ (Supplementary Fig. S8). Two docking interactions are particularly noteworthy. Asp127, located near the COXIIA C-terminus, and Arg19, located near the COXIIB N-terminus, have been predicted to form the only docking ionic bond in *D. tejensis* sp. nov. The second interaction involves the formation of hydrogen bonds between the supernumerary Thr96 in COXIIA, found only in *Dielis*, and Leu81 of COXIIB (Fig. 5c, Supplementary Fig. S8). In the COXIIA-COXIIB docking decoy taken out of the complex IV context, COXIIB is somewhat rotated versus COXIIA compared to the orientation of their corresponding regions in the crystal structures of canonical COXII in complex IV. However, this difference in orientation may be functionally nonsignificant or adjusted during complex IV assembly. Interestingly, two hydrophobic residues flanking metal-binding antiparallel β-sheets have been substituted in *Dielis* COXIIB by Cys residues. They have the potential to form a disulfide bond that may affect the folding and functioning of the Cu_A_ centre in a reduction/oxidation (REDOX)-dependent manner.

No extended similarities between the *cox2*-splitting insert and other parts of the mitogenome were found by BLAST that would suggest *cox2* fission due to, e.g., segmental duplication followed by complementary partial degeneration of the resulting gene copies. The entire 2.5-kb insert is on average 10% less AT-rich than the rest of the mtDNA and 0.5 kb shorter than the corresponding region in the *D. p. fossulana* mitogenome. Its L strand contains 1.4-kb- and 1.2-kb-long, partially overlapping open reading frames (ORFs) extending from *cox2a* or into *cox2b*, respectively (Supplementary Fig. S9). Despite carrying numerous nucleotide substitutions and indels, the former ORF retained similarity to the hypothetical *qnu* gene of *D. p. fossulana* that was proposed to encode, at least in the past, a nuclease-like protein^8^. The H strand carries a 1.1-kb-long ORF antisense overlapping with the *qnu* ORF. Interestingly, the 5’- and 3’-regions of the insert exhibit traces of mutual sequence similarity (45% identity, E < 0.3e−1) (Supplementary Fig. S10), which together with the possibility of a link between QNU and nucleases seems to further support the mechanism of *cox2* fission by insertion of a transposable element.

### Loss of *cox2* integrity in *Megacampsomeris*

The identification of yet other Scoliidae genera with split *cox2* gene is essential for the timing of evolutionary *cox2* fission in wasps. Recently, the mitogenome sequence of *Megacampsomeris prismatica* (Smith), an Indomalayan representative of another major genus of Campsomerini, was made available in the GenBank database (MH748671) and was analysed here for the structure of the *cox2* locus. BLAST analysis of conceptually translated *M. prismatica* mitochondrial ORFs revealed homology of one of them to *Dielis cox2a* gene but did not recover the remaining essential 3’ half of *cox2*. Based on multiple sequence alignment of *cox2* genes from *Dielis* and other Hymenoptera, the *M. prismatica cox2a* termination codon is either abbreviated and posttranscriptionally completed as in *Dielis* by polyadenylation or is located further downstream, resulting in an enlarged COXIIA protein. The sequence of *M. prismatica* mtDNA, downstream of *cox2a* homology region, is 38% identical (E < 9.9e−5) to the *cox2*-splitting insert in *Dielis* mitogenomes, suggesting the involvement of a gene splitting event common to both genera. It also suggests that the *cox2a* 3′-end may be generated posttranscriptionally, similar to that in the genus *Dielis*. A fossil specimen attributed to *M. prismatica* is known from the early Miocene formation dated at 16–20 million years (My)^44^. It is assumed that clades leading to *Dielis* and *Megacampsomeris* diverged 10–20 My earlier in the second half of the Palaeogene, and the Campsomerini and Scoliini lineages diverged in the late Cretaceous^26^. Thus, *cox2* fission in Scoliidae may have occurred around the turn of the Mesozoic and Cenozoic eras.

### Uniqueness of the *cox2*-splitting insert and frequent size increase of mtDNA in Hymenoptera

**Figure 6:**
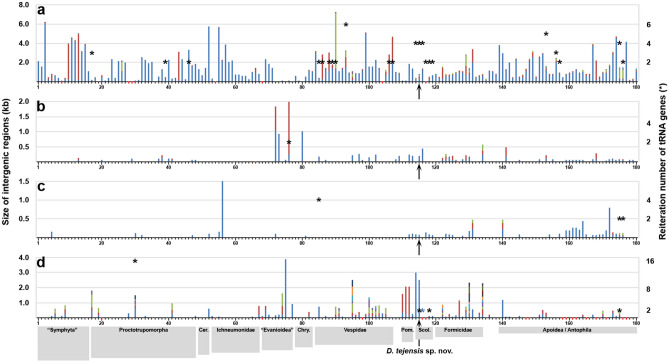
Increased intergenic distance and *trn* copy number in the mitogenomes of Hymenoptera. The 180 species analysed here are listed in Supplementary Table S4 together with additional details on the plotted data. Red marks on the x-axis indicate no data from a given mtDNA region. The graph bars represent noncoding regions larger than 50 bp. Different bar colours depict subregions of larger regions interrupted by *trn* genes/pseudogenes. Abbreviations: Cer., Ceraphronoidea; Chry., Chrysidoidea *s. lato*; Pom., Pompiloidea; Sco., Scoliidae. The following mitogenomic regions were analysed: (**a**) *rrnS-nad2* or adjacent to at least one of these genes (including the CR); (**b**) *nad2*-*cox1*; (**c**) *cox1*-*cox2*; (**d**) *nad3*-*nad5* and/or other intervals. Asterisks denote the copy numbers of the following tRNA genes characterized by high covariance score and/or homology to a functional *trn*: (**a**) *trnQ*, *trnI*, *trnS1*, *trnM*, or *trnL1*; (**b**) *trnE*; (**c**) *trnL2*; (**d**) *trnF*, *trnN*, or *trnK*. The blue asterisk indicates that the *trn* duplicate has been translocated into another *trn* cluster.

The possibility of *cox2* splitting occurring through ancient intragenic insertion of a mobile element prompted a survey of the hymenopteran mitogenomes for potential evidence of other insertions of extramitochondrial origin. At least one species from each tribe/subfamily/family represented in the GenBank database was taken into account including all species with mitogenomes larger than the average bilaterian mtDNA. This analysis showed that although the largest CRs are present in Symphyta and some aculeate genera (e.g., *Bombus*), enlargement of other noncoding regions was frequently seen in all groups of Apocrita (Fig. 6). The intergenic regions that were enlarged most often and to the greatest extent included those also enlarged in *D. tejensis* sp. nov. Nevertheless, repeat finder and phylogeny-wide BLAST analyses of those intervals showed that even the largest of them arose through duplication of adjacent, usually short mitochondrial sequences, including duplications of the *trn* genes (Fig. 6). The majority of *trn* copy number changes were found in the *trn* gene cluster typically located between the CR and *nad2*. Of 10 duplicated *trn*s in Hymenoptera, the highest reiteration numbers, six and five, have been determined for *trnS1* in the proctotrupoid wasp *Vanhornia eucnemidarum* (GenBank_DQ302100) and *trnL1* in the bee *Apis andreniformis* (GenBank_KF736157), respectively. The most frequently duplicated (in both Parasitica and Aculeata) was *trnM*. No duplicated *trn* genes were found between PCGs in Symphyta.

## Discussion

The new *Dielis* species described here increases the representation of the group of Scoliidae wasps featuring a split *cox2* gene and its protein product. Although the morphological differences between males of *D. tejensis* sp. nov. and the closest related sympatric species *D. p. fossulana* are rather minor, as is the case with other *Dielis* species, the differences in colour pattern, which exhibit relatively low intragenic variability in this genus, are noticeable. The taxonomic value of the colour pattern in *Dielis* was already mentioned by Bradley^30^ while defining the *D. plumipes* group. In fact, it remains one of the major external traits on which identification keys to *Dielis* species are based. The *D*. *tejensis* sp. nov. female has yet to be found, and may not differ significantly in phenotype from females of the sibling species, or there may be a disparity between the abundances of the sexes similar to that reported for *D. plumipes* in its eastern range^30^. The largest differences between the mitogenomes of *D. tejensis* sp. nov. and *D. p. fossulana* relate to *trnK*^*ttt*^ copy number and size of some intergenic regions. The 6.6% distance between the barcode *cox1* genes of these two species is approximately 30 times greater than their intraspecific variability, reaching the level of *cox1* divergence often observed between hymenopteran sibling species. Based on the *cox1* clock rate, recalibrated at a 3.5% sequence divergence in My^45^, the lineages of *D*. *tejensis* sp. nov. and *D*. *plumipes* may have remained separate for the last 2 My. This estimate is in agreement with the time of the *Dielis* radiation proposed, based on the phylogenetic analysis of ultraconserved nuclear genome elements as taking place in the last 4.5 My^26^. Thus, the origin of *D.* *tejensis* sp. nov. and related species may be linked to early Pleistocene glaciations that displaced North American fauna to isolated Central and South American refugia, creating barriers to gene flow.

**Figure 7:**
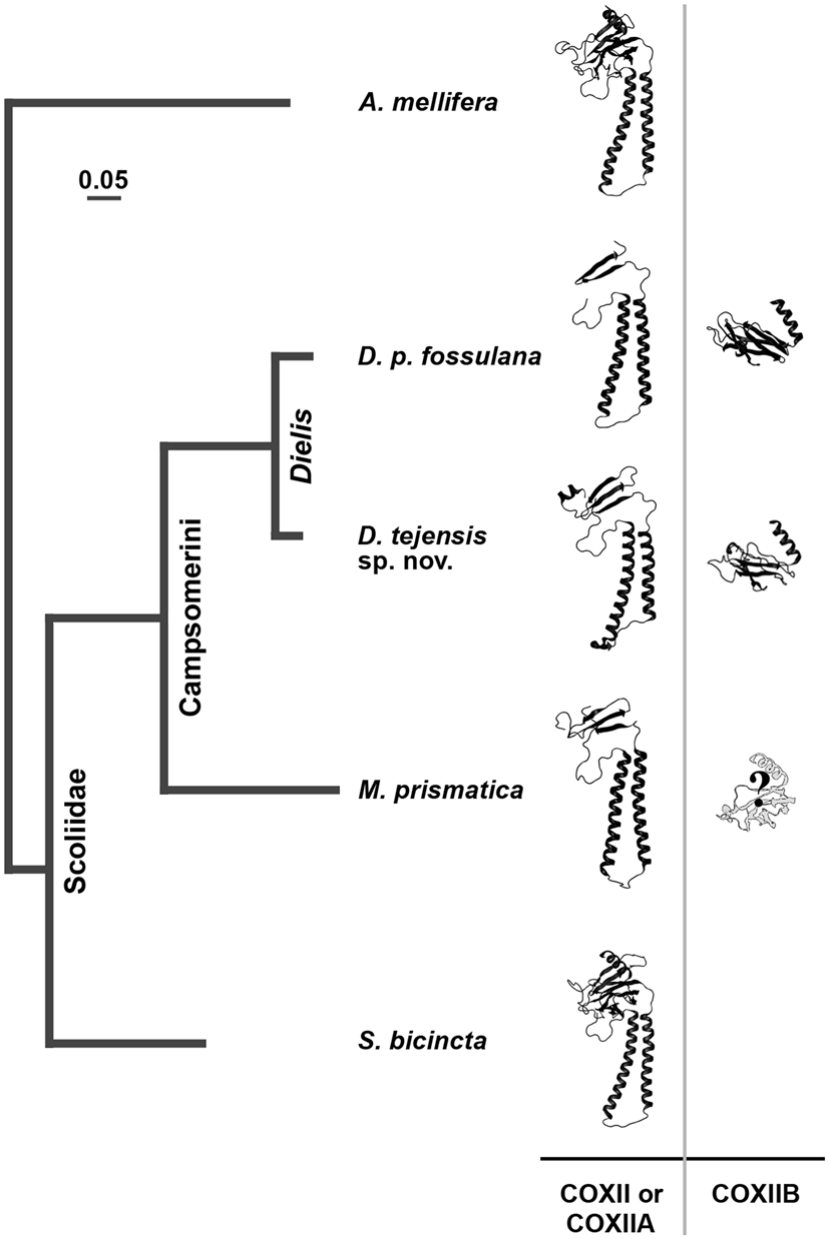
Graphical synopsis of the evolution of *cox2* dimorphism in Scoliidae. The phylogram depicts the maximum likelihood genealogy of the *cox2a* gene or a corresponding region in undivided *cox2* (GTR distance). The “?” refers to the unknown location of the *cox2b* gene.

The fission of mitochondrial *cox2* apparently preceded the origin of the genus *Dielis* since it is now also reported in *Megacampsomeris*. In the context of mitogenome evolution, it is surprising that the *M. prismatica cox2b* gene could not be identified. While the possibility of mtDNA sequence incompleteness cannot be ruled out, *cox2b* could also be translocated to the nucleus. Compared to COXIIA, COXIIB is much less hydrophobic and thus less likely than COXIIA or full-length COXII to be redirected to the endoplasmic reticulum if translated outside mitochondria (e.g., Ref.^46,47^). In agreement with these predictions, in chlorophycean algae, in which at least one of the *cox2*-derived genes has been transferred to the nucleus, *cox2b* was always the relocated gene^22^. The current understanding of the evolution of *cox2* gene dimorphism in Scoliidae is summarized in Fig. 7.

The split of *cox2* seems to be causatively linked to additional structural changes in COXIA and COXIIB proteins. Some of them, such as the insertion of Thr in *Dielis* COXIIA, likely contribute to COXIIA-COXIIB docking; others, such as amino-acid substitutions with REDOX-sensitive Cys around the Cu_A_ centre, may have increased the responsiveness of complex IV to oxidative stress, which likely followed the gene fission itself. Of note, the environment of the mitochondrial intermembrane space does enable the formation of disulfide bonds^48^.

The mechanism of *cox2* fission in wasps remains unknown, but the available data seem to suggest the involvement of transposon insertion. Nevertheless, integration of a mobile element into animal mtDNA would be a rare event, at least since the appearance of arthropods, and only recently has it been proposed to occur during the evolution of ticks^49^. The interruption of *cox2* in bivalves has been suggested to result from insertion of a selfish viral element^12^. In many groups of land plants, mitochondrial *cox2* is interrupted by retrotransposon-derived group II introns often inserted between the transmembrane and intermembrane domain-coding regions, similar to the inserts in wasps, bivalves and protists (reviewed in, e.g., Ref.^50^).

Interestingly, not only have the mitogenomes carrying the *cox2*-splitting insert not been eliminated over the course of evolution, but the size of some other intergenic regions in mtDNA of Campsomerini and other Hymenoptera also increased compared to those in the majority of the remaining insects. In particular, the *nad2-trnW* interval, although much smaller than *cox2a-cox2b*, is specifically enlarged in *D. tejensis* sp. nov. mtDNA. It may harbour alternative O_R_(s), and due to its repetitive nature, it likely originated by slipped-strand mispairing followed by point mutations and indels. Its syntenic region in vertebrate mtDNA does harbour the origin of lagging strand replication^51^. A secondary O_R_ was proposed to be located between the *cox1* and *cox2* genes in the honeybee *Apis mellifera*^52,53^, in a region that is also enlarged in *Dielis*, and between *trnS2* and *nad1* in *Triatoma dimidiata* (Hemiptera)^54^. Of note, some of the largest noncoding regions in insect mitogenomes map, as in *D. tejensis* sp. nov., between the *nad2* and *trnW* genes and reach up to 6.5 kb in seed beetles (Coleoptera)^55^, 2.9 kb in earwigs (Dermaptera)^56^ and 1.8 kb in katydids (Orthoptera) (GenBank_NC034756). As in *D. tejensis* sp. nov., these intervals are apparently the result of tandem duplications of smaller mtDNA sequences. The increased presence of enlarged mitochondrial intergenic regions may seem to be the rule in apocritan Hymenoptera, some families of other insect orders, molluscs and nematodes, but is not in line with the general trend in bilaterian animal evolution towards minimization of mtDNA size through the elimination of intergenic regions in the first place (reviewed in, e.g., Ref.^57^).

## Conclusion

Based on mitogenome analyses of the described here apparently endemic to Texas new species, *D. tejensis*, other Scoliidae and representatives of other hymenopteran families, it seems that the unique for animals organization of the *cox2* locus, featuring two complementary genes encoding both halves of the conventional COXII, may be limited to Scolidae tribe Campsomerini. The current stage of *cox2* evolution in Scoliidae resembles to some extent the evolution of this gene in green algae, in some of which it has a canonical structure, whereas in others, it is divided into *cox2a* and *cox2b* genes, with *cox2b* and in some cases also *cox2a* being translocated to the nuclear genome. Another intriguing feature of the mtDNA of *D. tejensis* sp. nov. and apocritan Hymenoptera in general is the frequent presence of enlarged intergenic regions. In particular, the *cox2*-splitting insertion is one of the largest unconventional loci found in the mtDNA of any bilaterian animal. This further shows that bilaterian mitogenomes, despite their compactness, can tolerate a certain increase in size and renews the question about the potential regulatory function of these regions. From yet another perspective, rearrangements, insertions and other discrete characters of the mitochondrial genomes of Scoliidae, beyond their potential importance for studies on the regulation of mtDNA expression, could also be leveraged to infer still incompletely recognized phylogenetic relationships within these economically beneficial and striking insects.

## Methods

### Taxon sampling

The Hymenoptera used here were either newly collected for this project in Southeast Texas or were museum specimens on loan from the Texas A&M University (TAMU) Insect Collection at College Station (Texas), the Louisiana State Arthropod Museum (LSAM) in Baton Rouge (Louisiana) and the Florida State Collection of Arthropods (FSCA) in Gainesville (Florida). The type specimens of *D. tejensis* sp. nov. have been deposited at TAMU and FSCA. Morphological nomenclature conforms to that recommended by the Hymenoptera Anatomy Ontology project^58^.

### Mitogenome sequencing

DNA was extracted from wasp legs using the NucleoSpin Tissue Kit (Macherey–Nagel, Düren, Germany). The entire mitogenome of newly caught specimens of *D. tejensis* sp. nov. was amplified in two overlapping fragments as described in Ref.^8^, using two primer pairs: mHCO2198/HPK16bb and ouCO2198/ou16S. Long-range PCRs were performed using LA Taq DNA polymerase (Takara, Ōtsu, Japan) and applying 30 cycles of incubation at 94 °C for 30 s and at 62 °C for 9 min. The two PCR-amplified fragments of the mitogenome were directly Sanger sequenced by applying a primer walking strategy. Mitogenomes were assembled using Sequencher (Gene Codes, Ann Arbor, Michigan). In addition, the intergenic region *nad2-trnW* and a fragment of *cox1* were amplified by regular 3-step PCR from 10- to 60-year-old pinned Scoliidae specimens using the primer pairs nd2sp/trnWsp and co1F1/co1R2, respectively (sequences of PCR primers are shown in Supplementary Data S2).

### Sequence analyses

tRNA-coding genes were found using tRNAscan-SE (https://www.trna.ucsc.edu/tRNAscan-SE), ARWEN (https://mbio-serv2.mbioekol.lu.se/ARWEN/index.htlm) and MITOS (https://mitos.bioinf.uni-leipzig.de/index.py). rRNA-coding genes and PCGs were identified by searching for BLAST similarities to reference genes (https://www.blast.ncbi.nlm.nih.gov/Blast.cgi). An ATN codon nearest to the preceding gene was designated as the initiation codon. Predicted abbreviated termination codons were experimentally verified following the 3′ RACE protocol (Clontech, Mountain View, CA). To this end, RNA was isolated from the legs of *D*. *tejensis* sp. nov. using the miRNeasy Mini Kit (Qiagen, Frederick, MD) and, following conversion to cDNA, mRNA 3′-ends were PCR-amplified for Sanger sequencing using gene-specific and UPM primers. Pairwise comparisons were performed in BLAST and Lalign (https://www.ebi.ac.uk/Tools/psa/lalign). Transcriptional promoters were predicted based on their similarity to human mitochondrial promoters^33,34^ and by using the NNPP program (https://www.fruitfly.org/seq_tools/promoter.html). Tandem repeats were identified with the Tandem Repeat Finder web server (https://tandem.bu.edu/trf/trf.htlm). Stem-loop structures were predicted using the mfold server accessible through the UNAFold portal (https://www.unafold.org). The relative synonymous codon usage (RSCU) was determined using CAIcal (https://genomes.urv.es/CAIcal). The *cox1*-based genetic distance between *D. tejensis* sp. nov. and *D. p. fossulana* was estimated in PhyML under a generalized time-reversible (GTR) substitution model using NGPhylogeny (https://ngphylogeny.fr).

### Protein three-dimensional structure prediction

COXII, COXIIA and COXIIB tertiary structures were modelled using the I-TASSER algorithm included in the NovaFold application run through Lasergene’s Protean 3D interface (DNAStar, Madison, WI). The alignment of protein tertiary structures was performed with Protean 3D. Protein docking interactions were explored utilizing the structure-based SwarmDock algorithm included in the NovaDock application run through Protean 3D.

### Nomenclatural acts

The electronic version (PDF) of this article constitutes a published work as defined in the amendment to the International Commission on Zoological Nomenclature (ICZN). The new name introduced here is effectively published under that code from the online edition of this article alone. This published work and the nomenclatural act that it contains have been registered in ZooBank (https://zoobank.org), the online registration system for the ICZN. The Life Science Identifier (LSID) for this publication is urn:lsid:zoobank.org:pub:7B25D0DA-2F0C-42B3-90F7-E7B6366B75B8. The article has been archived and is available from digital repositories, including Nature Research and PubMed Central.

## Supplementary Information

Supplementary Information.

## Data Availability

Reported here mitogenome sequences are deposited in the NCBI GenBank database (https://ncbi.nlm.nih.gov/genbank) under accession number MN990424.
