# Supplementary material for: New Dielis species and structural dichotomy of the mitochondrial cox2 gene in Scoliidae wasps
Source: Sci Rep. 2023 Feb 2;13:1950. doi: 10.1038/s41598-023-27806-x (PMC9895450; doi:10.1038/s41598-023-27806-x)
Supplement: Supplementary file 1 — Supplementary Information. [file 41598_2023_27806_MOESM1_ESM.pdf]

SUPPLEMENTARY INFORMATION

New *Dielis* species and structural dichotomy of the mitochondrial *cox2* gene in Scoliidae wasps

Przemyslaw Szafranski

Department of Molecular and Human Genetics, Baylor College of Medicine, One Baylor Plaza, Houston, Texas 77030, USA

Supplementary Data S1. List of *D. tejensis* sp. nov. paratypes.

Paratype males: USA, Texas, [Bastrop Co.], McDade, 26/IV/1934, J. E. Gillaspy leg. (TAMU); USA, Texas, Bastrop Co., 15/VII/1935, J. E. Gillaspy leg. (TAMU); USA, Texas, Bastrop Co., 22/IV/1973, J. C. Schaffner leg. (TAMU); USA, Texas, Bastrop Co., near Bastrop, 13/VII/1985, D. Heffern leg. (*cox1* sequenced) (TAMU); two specimens, USA, Texas, Kent Co., near Jayton, 4/VII/1989, J. C. Cokendolpher leg. (*cox1* sequenced) (FSCA); USA, Texas, Bastrop Co., Sayera, 19/III/1992, McGuirt leg. (TAMU); USA, Texas, [Willacy Co.], 18/III/1994, W. F. Chamberlin leg. (TAMU); USA, Texas, Kenedy Co., 6-20/IV/2001, W. Godwin & E. Riley leg. (TAMU); USA, Texas, Harris Co., Houston, 15/X/2013, P. Szafranski leg. et col. (*cox1* sequenced); USA, Texas, Harris Co., Houston, IX/2014, P. Szafranski leg. et col. (*cox1* sequenced).

Supplementary Data S2. PCR primers used to amplify Scolidae mtDNA (5'>3' sequences).

>mHCO2198
TAAAATATAAACTTCAGGGTGWCCAAAAAAYCA

>HPK16bb
CTTATCGAYAAAAAAGWTTGCGACCTCGATGTT

>ouCO2198
GTAGGAAAAGGAATTGGAACTGGATGAACTA

>ou16S
GAATAATGACATCCTGAAGATCAGCCAGAA

>nd2sp
ATTGCYAYWTCWATATTTCCATATTTATTTTAT

>trnWsp
TTTGATTATTACAATTTTAACTTTGAAGG

>co1F1
TCGTATAGAACTAGGAATAGCAGGATC

>co1R2
TCCAGTTCCAATTCCTTTTCCTACA

Supplementary Data S3. Detailed description of *Dielis tejensis* sp. nov. male.

Size

Body length 17.8 ± 2.1 mm (SD); fore wing length 14.3 ± 1.5 mm (SD) (n = 10)

Morphology

Head, antennae and mouthparts: Vertex immediately behind the ocelli only with scattered punctures but becomes densely punctate posteriorly; gena much more densely punctate, posteriorly like vertex; frons with a central pit, largely impunctate below the anterior ocellus, laterally with two weakly defined punctate glabrous tubercles with bristles arising from pits; frontal spatium densely punctate except small space immediately below the central pit; frontal area irregularly and somehow less densely punctate; clypeus broadly punctate laterally and basally with long bristles arising from the pits and with one or two rows of punctures along its apical margin, disk of clypeus almost impunctate with weak irregular lengthwise wrinkles; hypostomal carina without traces of submandibular triangle. Antenna with 13 segments reaching the apex of propodeum, scape and pedicel punctate and covered with bristles. Mandibles with three very weakly expressed centrally located teeth, length of the maxillary palp equal to approximately one-fourth of the length of the hypostoma.

Mesosoma, wings and legs: Anterior vertical part of the pronotum mostly impunctate, dorsal posterior and the upper half of the lateral vertical areas of pronotum densely punctate, the lower narrowing half with only very few dispersed small punctures; anterior half of mesopleuron punctate except its anterior-most third part, posterior half very sparsely punctate with large impunctate areas; dorsal aspect of the upper plate of metapleuron densely punctate, lateral aspect of the upper plate almost impunctate, lower plate sparsely punctate with weak punctures obscured by appressed pubescence; mesoscutum punctate but somewhat less densely than pronotum; scutellum with upper portion punctate more or less as mesoscutum but broadly impunctate along the posterior margin; metanotum punctate except the posterior third part; propodeum in dorsal view with subhorizontal portion approximately one-third the basal width, not rounding immediately behind the scutellum, making an obtuse angle with the posterior vertical part, lateral carina of propodeum discontinuous beyond the spiracle, spiracular angle gradually less punctate to impunctate towards the spiracle, dorso-median and dorsolateral areas densely punctate, lateral surface very sparsely punctate and not striate. Tegulae featuring only very few punctures at their base. Forewings with two submarginal cells and two recurrent veins (second recurrent vein connected to the second submarginal

cell), setose on anterior one-third, mostly bare beyond the cells. Spurs of the hind tibiae unequal in length, inner spur spatulate.

Metasoma and terminalia: The median-length-to-width ratio of the 1st tergite (excluding stalk) is 0.88 ± 0.06 (SD). In comparison to *D. p. fossulana*, this ratio is approximately 12% higher (*t*-test $P < 0.05$); tergites densely punctate except the basal margins which are largely impunctate or have only very scarce fine punctures; 1st sternite almost impunctate, other sternites punctate as tergites but with very minute punctation on their basal third up to half; gradulus absent. Genital capsule (Fig. 2b) (n = 2): volsellae consisting of two parts, the digitus and cuspis; a distal segment of the gonopode, the paramere (harpe), wider than those in *D. p. fossulana* and *D. tolteca* (the ratio of length-to-width at base for the harpe 2.6 compared to 2.9 and 2.8 for *D. p. fossulana* and *D. tolteca*, respectively); length-to-width ratio for aedeagus blade 2.4, similar to that for *D. tolteca*, but 10% lower than that (2.6) for *D. p. fossulana*. Bristles on the ventral surface of the paramere in *D. tejensis* are shorter than those in *D. tolteca*. The sensory cones on the digitus are larger and occupy a larger area of the digitus than those in *D. p. fossulana* and *D. tolteca*.

Colouration: Body integument mostly black (abdomen with faint blue and greenish reflections) marked with yellow as follows: short stripe on the eye inner margin running from the ocular sinus down towards the malar space (Supplementary Fig. S2a); a stripe along the posterior margin of the pronotum abutting against spots on pronotal antero-dorsal corners (Supplementary Fig. S2b); moderately elongated spot on the callosity along the anterior margin of the vertical portion of the pronotum (Supplementary Fig. S2c), present in the majority of the examined specimens; in some cases a small spot at the posterior corner of the pronotum next to the tegula; scapulae immaculate; transverse stripe on the scutellar disc; a central spot on the metanotum; apical bands on 1st-5th tergites; centrally interrupted apical bands on 2nd-5th sternites (Fig. 2a). The apical band on the 1st tergite is slightly wider than the corresponding band in *D. p. fossulana* (Fig. 2a); the median notch on the band on the 2nd tergite is narrower than its counterpart in *D. p. fossulana* (Fig. 2a), although not acute like that in, e.g., *D. trifasciata*. Mandible black-brown, yellow basally (Supplementary Fig. S2a). Antenna black-brown with brown scape. Legs mostly black with the following yellow markings: a small spot on the apex of the femora; yellow line on the outer side of the front tibia (Supplementary Fig. S2d), extending through almost the entire basitarsus and through up to one-third of the femur; in half of the examined specimens, a small spot on the anterio-lateral side close to the apex of meso- and metafemur. Protarsi brownish. Front tibial spurs

brown, metatibial spurs white. Tegula translucent and brown with a basal yellow spot (Supplementary Fig. S2b-c). Wing membranes hyaline and slightly yellow-brownish, more infumated with brown along the anterior margin; wing setae and veins brown (Fig. 1, Supplementary Fig. S1). Vestiture mostly white, white and black on 5th metasomal segment and black on 6th and 7th segments.

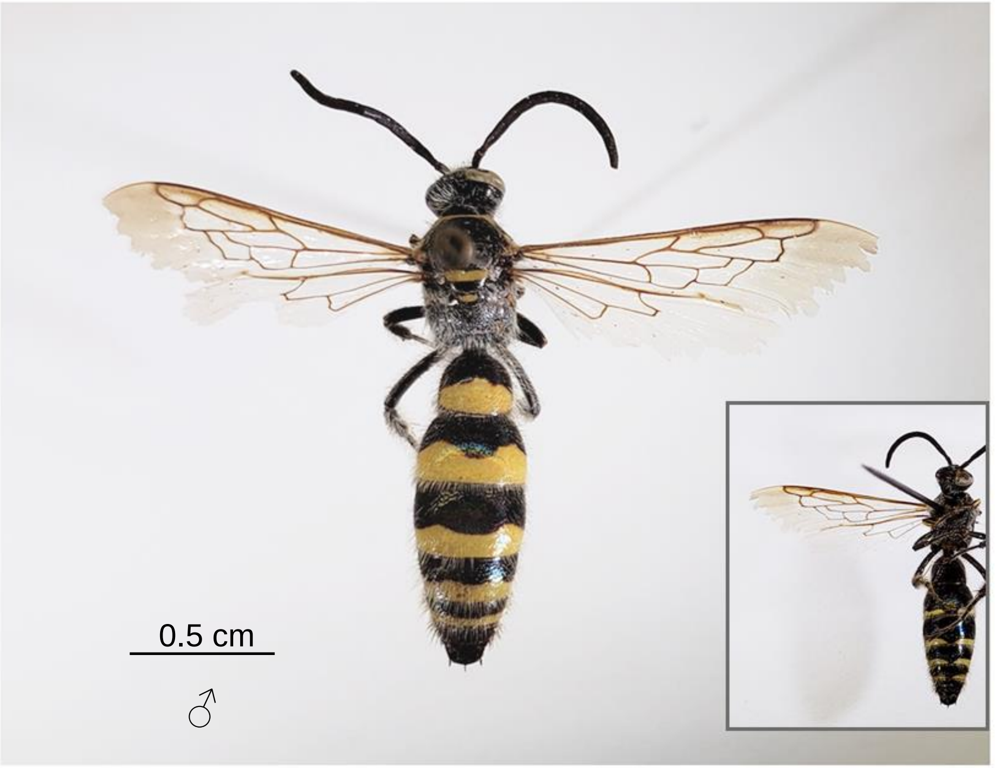

Supplementary Figure S1. *D. tejensis* sp. nov. holotype male, dorsal and ventral (inset) habitus.

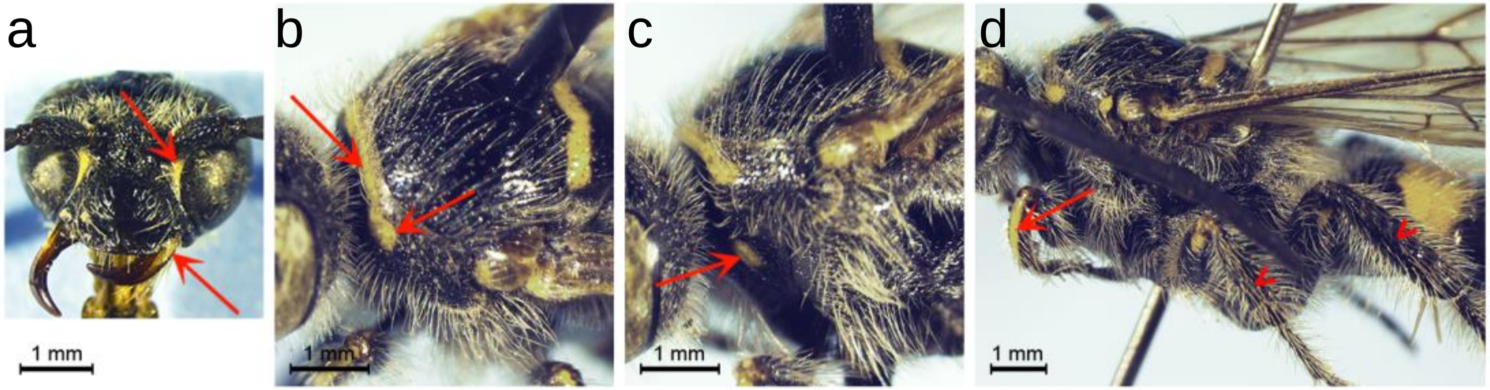

Supplementary Figure S2. Additional phenotypic features of *D. tejensis* sp. nov. (**a**) Head, (**b**,**c**) mesosoma, (**d**) tibiae. Red arrows point to the discussed yellow markings at the base of the mandibles and on the eye margin (a), pronotum (b,c) and outer side of the front tibia (d). Arrowheads point to the absence of yellow strips on meso- and metatibiae.

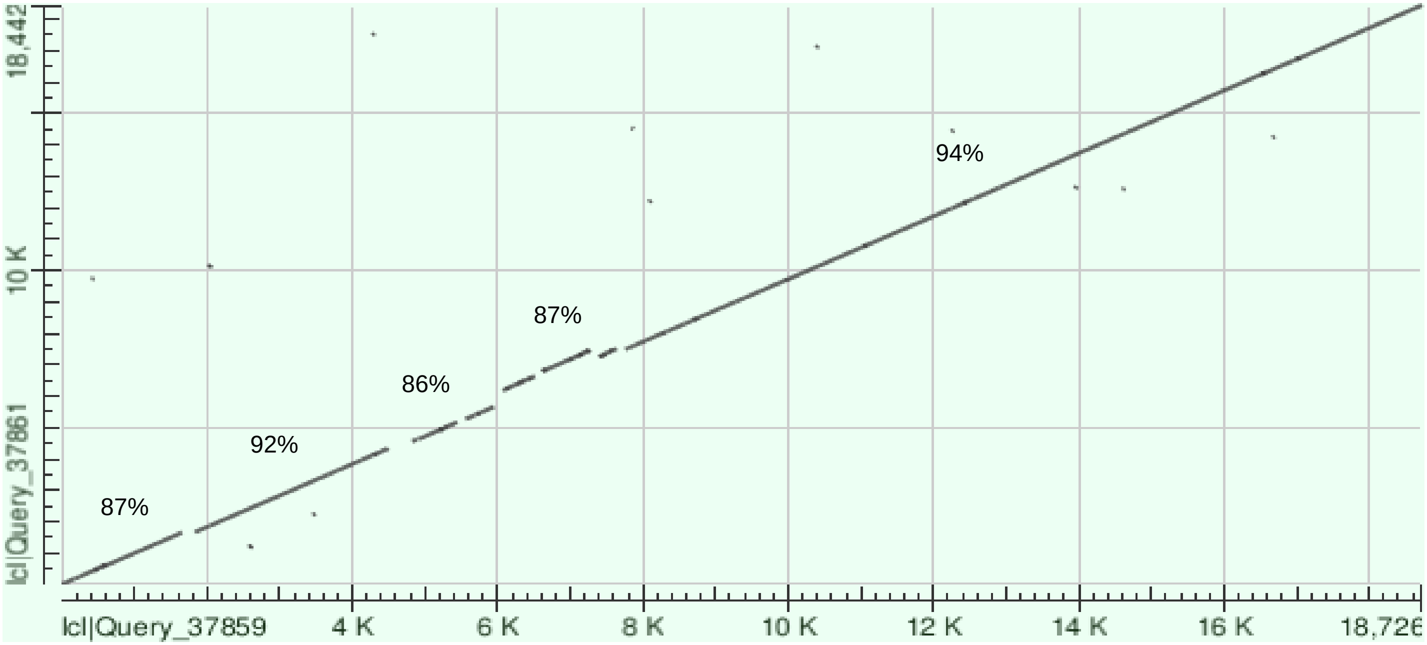

Supplementary Figure S3. Dot plot of the pairwise alignment in BLAST of *D. tejensis* sp. nov. (X-axis) and *D. p. fossulana* (Y-axis) mitogenomes. The indicated percentage of identity applies to diagonal lines of 1 kb or longer sequence matches between the two species.

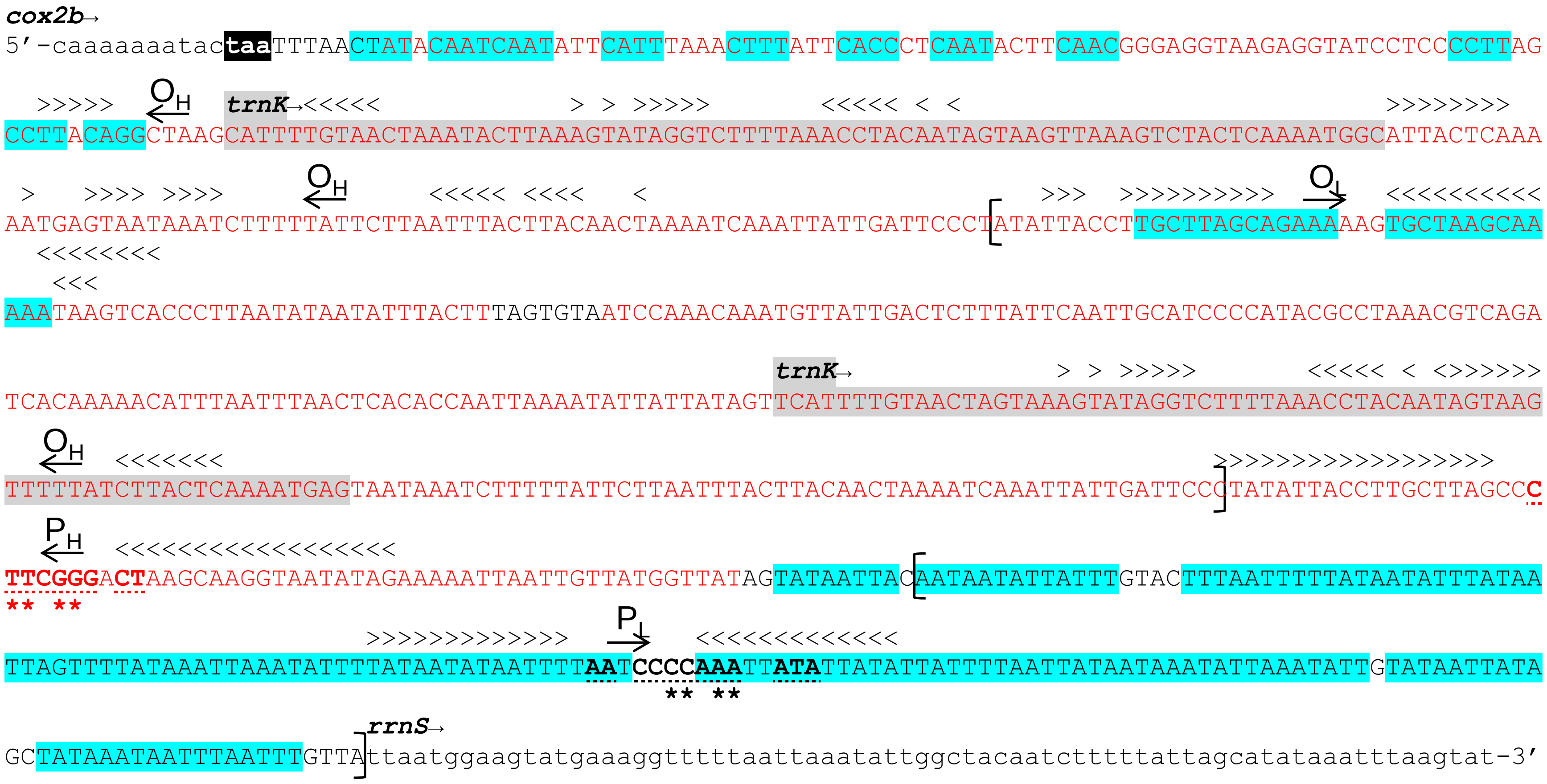

Supplementary Figure S4. The expanded regulatory region (CR) of *D. tejensis* sp. nov. (mtDNA L strand). Flanking sequences of *cox2b* and *rrnS* are in lowercase letters. The *cox2b* stop codon is on black background. The *trnK*^{ttt} gene and its duplicate are highlighted grey. The 0.3 kb-large segmental duplication is shown in red. Short repeats are highlighted blue. Stem regions of mfold-predicted palindromes are indicated by strings of arrowheads. Regions in brackets are not represented in *D. p. fossulana* CR. Underlined sequences represent homologies to the consensus motif of human mitochondrial promoters[32]. Asterisks indicate bases corresponding to those interacting in human mitochondria with TFB2M (non-template strand) and mtRNAPol (template strand)[33]. O_R, putative origin of replication; $P_{H/L}$, putative promoters.

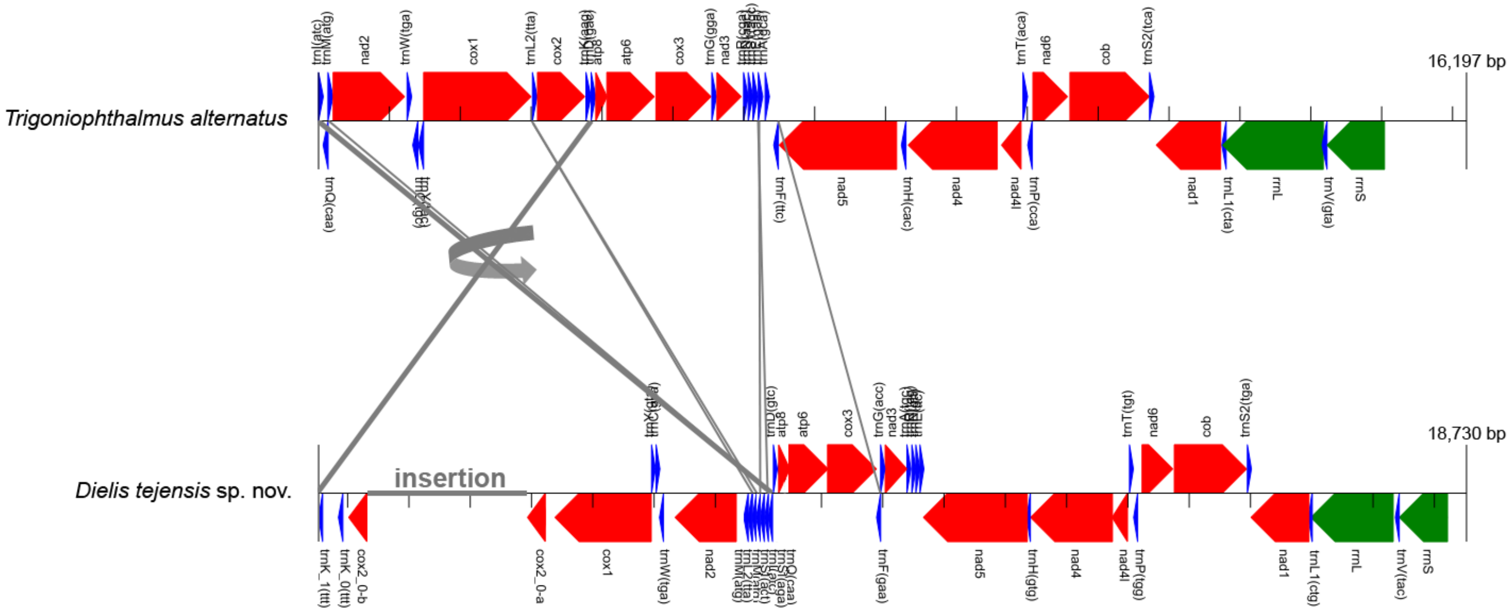

Supplementary Figure S5. Mitochondrial gene rearrangement in *D. tejensis* sp. nov. The presumably ancestral organization of the insect mtDNA is illustrated by that of *Trigoniophthalmus alternatus* (Archaeognatha). Diagrams of the mitogenomes have been arbitrarily interrupted. Codons recognized by tRNAs are given in parentheses next to a *trn* gene symbol.

a

```
trnM^cat    331 ATATAAATAGGCTAATATAATAAGCTTTTAGACTCATA-------ATTTAAAGATGAATAAT-TATTCTTTA 398
                 **** *** * *** ***  ** **  **********       * *   **** ** * * * **** **
trnM^cat    465 GTATAGATAAGTTAAAATACAAAACTACTAGACTCATACCCTAGCAATAGCAGATTAAAACTCTCTTCTATA 538
```

b

```
trnM^cat    331 TATAAATAGGCTAATATAATAAGCTTTTAGACTCATAATTTAAAGATGAATAATTATTCTTTATATA 398
                * ***   ***  *  ** *  **  * *** * *   * ** * *  ** **   * ****   **
trnL2^taa   401 TTTAATATGGCAGAGTTAGT--GCAAT-AGATTTAAGTTCTATAAACAAAGAAAATTACTTTTATTA 464
```

c

```
trnS1^tct   136 GAAATAAA--AAAAGTTAGATTTCTAAT---CTAATAATTAATTATTAATAATAATTATTAT 196
                **  ****  ******** *** *****   ****  * ** **  ** *****  * * **
trnS1^act   268 GAGGTAAATTAAAAGTTA-ATTACTAATTAACTAAGTAATATTTTATATTAATATCTTTAAT 333
```

d

```
trnK^ttt  7,080 CATTTTGTAACTAAATACTTAAAGTATAGGTCTTTTAAACCTACAATAGTAAGTTAAAGTCT-ACTCAAAATG 7,152
                *************      ************************************    *** **********
trnK^ttt  7,410 CATTTTGTAACTAG-----TAAAGTATAGGTCTTTTAAACCTACAATAGTAAGTTTTTATCTTACTCAAAATG 7,478
```

Supplementary Figure S6. Homology assessment of supernumerary *trn* genes of *D. tejensis* sp. nov. Pairwise sequence alignment in Lalign of (**a**) *trnM*^{cat}s, (**b**) *trnM*^{cat} (coordinates 331-398) and *trnL2*^{taa}, (**c**) *trnS1*^{tct} and *trnS1*^{cta}, (**d**) *trnK*^{ttt} genes. Asterisks indicate nucleotide identities.

a

***D. tejensis* sp. nov.**

TTTAAA**TAA**acaaataatagtttataaactaaacattaaattttaatttaataatgtttcgttattt**ATG**AAGCTATTTGTAGTGTACCAATGAAAAATAATAAATTTTCAAGACCCCG

cox1 ***cox2a***

D. p. fossulana

TTTAAA**TAA**acaaataatagtttattaattaaacattaaattttaatttaataatgctttgttatcttatgaagctatttgtg**ATG**TTTCAATGAAAAATAATAAATTTTCAGGATCCA

cox1 ***cox2a***

b

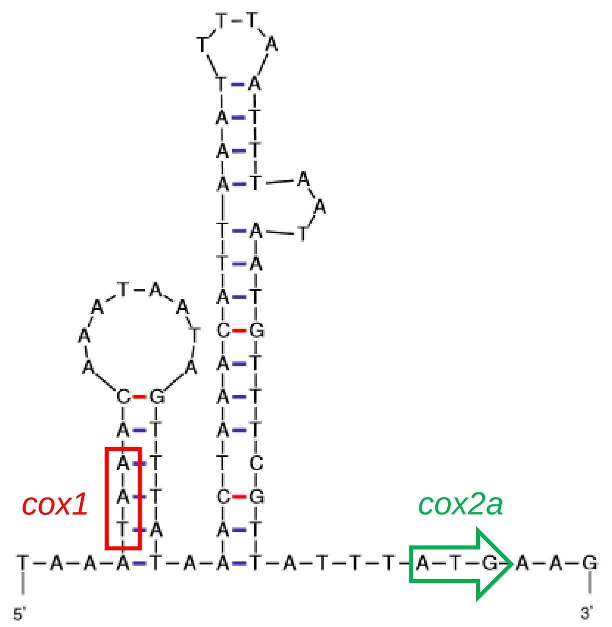

Supplementary Figure S7. Comparison of the *cox1*-*cox2a* intergenic region of *D. tejensis* sp. nov. (*Dtj*) and *D. p. fossulana* (*Dpf*) (mtDNA L strands). (**a**) DNA sequence alignment. Flanking coding sequences are highlighted in yellow. (**b**) Predicted folding of the *D. tejensis* sp. nov. *cox1*-*cox2a* region in the single-strand conformation (mfold dG = -7.82 kcal/mol).

COXIIA or COXII

```
Polytomella sp. COIIA    TASAEAPVAW-QLGFQDSATSQAQAAFDLHHDIFFFLLNTVVLVFYFLYHIATKFHYTKQ   185
D.tejensis COXIIA        -MKLFVVYQWKMMNFQDPVSPNMQAMIGFHDLIMCITITIIILISYNYYFIS-----NNG   54
D.p.fossulana COXIIA     ------MFQWKMMNFQDPVSPNMQAMIGFHDLIMCITIMIIILIVYNYYFIS-----NNG   49
S.bicincta (COXII)       ------MNQWLHLNFQDPVSPNMEAMIYFHDFLNMILMMIMIIILYNFIIIF-----MNK   49
A.mellifera (COXII)      ------MSTWFMFMFQESNSYYADNLISFHNMVMMIIIMISTLTVYIILDLF-----MNK   49
D.melanogaster (COXII)   ------MSTWANLGLQDSASPLMEQLIFFHDHALLILVMITVLVGYLMFMLF-----FNN   49
                                  *     *             *               *
                                             αααααααααααααααααααααααααααααααααααααααα

Polytomella sp. COIIA    ALPEKLTHHTAIEVIWTVIPTIIVVLIAIPSLTLVYAMDSHNDKPGLTVKVIGRQWYWSY   245
D.tejensis COXIIA        YTYRKLTHGSFIEAIWTMLPIIILVLLSIPSMKILYMNDEGTLNPSMTVKVLGNQWFWTA   114
D.p.fossulana COXIIA     YTYRKLTHGSFIEAIWTMLPIIILVLLSIPSMKILYMNDEGTLNPSMTVKVMGNQWFWSA   109
S.bicincta (COXII)       LTFRYLINGHVLELIWTIIPIFILITIAIPSMKILYFTED-IFLPLMTIKMIGNQWFWIA   108
A.mellifera (COXII)      FSNLFLLKNHNIEIIWTIIPIIILLIICFPSLKILYLIDE-IVNPFFSIKSIGHQWYWSY   108
D.melanogaster (COXII)   YVNRFLLHGQLIEMIWTILPAIILLFIALPSLRLLYLLDE-INEPSVTLKSIGHQWYWSY   108
                              *    * * ***  *  *      **             *    *  * ** *
                                      ααααααααααααααααααααααααααα        βββββ    βββ

Polytomella sp. COIIA    EMHDHLQHKLLDADRLVAIAEKTITK----------------------------------   271
D.tejensis COXIIA        EYSGFDSNSMDFDMYLNKVLKEEQEKNGSFA-----------------------------   145
D.p.fossulana COXIIA     EYSGFD------------------------------------------------------   115
S.bicincta (COXII)       EYQDMIINKSFNNPLSIEMYMSSD------------------------------------   132
A.mellifera (COXII)      EYPEFNN--IEFDSYMLNYN----------------------------------------   126
D.melanogaster (COXII)   EYSDFNN--IEFDSYMIPTNE---------------------------------------   127
                         *
                         ββ       βββββ       ααααααα
```

COXIIB or COXII

```
Polytomella sp. COIIB    IQYNFDSYMVTDVQPGQLRMLEVDERLVLPTNTLVRLLVTASDVIHSWAVPSLGIKMDAI   99
D.tejensis COXIIB        ------MKYLKKKGKKLFRLLDTDRRMVVPINNPIRVLTSSLDVIHSFAIPSMGVKVDSV   54
D.p.fossulana COXIIB     ---------------MCFRLLDTDRRMVVPINNPIRILTSSLDVIHSFAIPSMGVKVDSI   45
S.bicincta (COXII)       ------------INKGMFRLLETDEHMVLPINSFIRLITTSEDVIHSFSLPSMGIKIDSV   180
A.mellifera (COXII)      -------------NLNQFRLLETDNRMVIPMKIPLRLITTSTDVIHSWTVPSLGIKVDAV   173
D.melanogaster (COXII)   ------------LMTDGFRLLDVDNRVVLPMNSQIRILVTAADVIHSWTVPALGVKVDGT   175
                                           * *  *   * *     *      *****   *  * * *
                                                                      βββββ    βββββ

Polytomella sp. COIIB    PGRLNQIWLTINREGVFYGQCSEICGANHSFMPIVVEAISPRAFLTEYVKKWIQ------   153
D.tejensis COXIIB        PGRLNQSFLYCQQMGLFFGQCSEICGLNHSYMPFCIEVTSYEKFM-----EWFKNIAKKY   109
D.p.fossulana COXIIB     PGRLNQSFLYCQQMGVFFGQCSEICGLNHSYMPFCIEVTNYENFL-----EWFKKVGKKY   100
S.bicincta (COXII)       PGRLNQSFMFSSQVGMFFGQCSEICGLGHSFMPITIEMVDKDTFL-----KWLIFKMMEN   235
A.mellifera (COXII)      PGRINQLNLISKRPGIFFGQCSEICGMNHSFMPIMVESTSFQYFL-----NWVNKQI---   225
D.melanogaster (COXII)   PGRLNQTNFFINRPGLFYGQCSEICGANHSFMPIVIESVPVNYFI-----KWISSNNS--   228
                         *** **        * * ********  ** **   *      *       *
                                       ββββββ            ββββββ  ααααα     ααααααααα
```

Supplementary Figure S8. Multiple alignment of COXII amino acid sequences of *D. tejensis* sp. nov., *S. bicincta*, the honeybee *A. mellifera* and the green alga *Polytomella* sp. Residues predicted to interact at the COXIIA/COXIIB interface in *D. tejensis* sp. nov. and *Polytomella* sp.[43] (Fig. 5c, Supplementary Table S4) are highlighted in green. The most conserved amino acid positions are indicated by an asterisk. Residues involved in metal ion binding are underlined. The Thr residue in COXIIA unique to the *Dielis*, its interactor Leu in COXIIB and the Asp-Arg pair of residues predicted to form a salt bridge between COXIIA and COXIIB are shown in red; unique Cys residues flanking metal-binding antiparallel β-sheets are highlighted in yellow. The α-helices and β-sheets refer to the secondary structures of *D. tejensis* sp. nov. COXIIA and COXIIB.

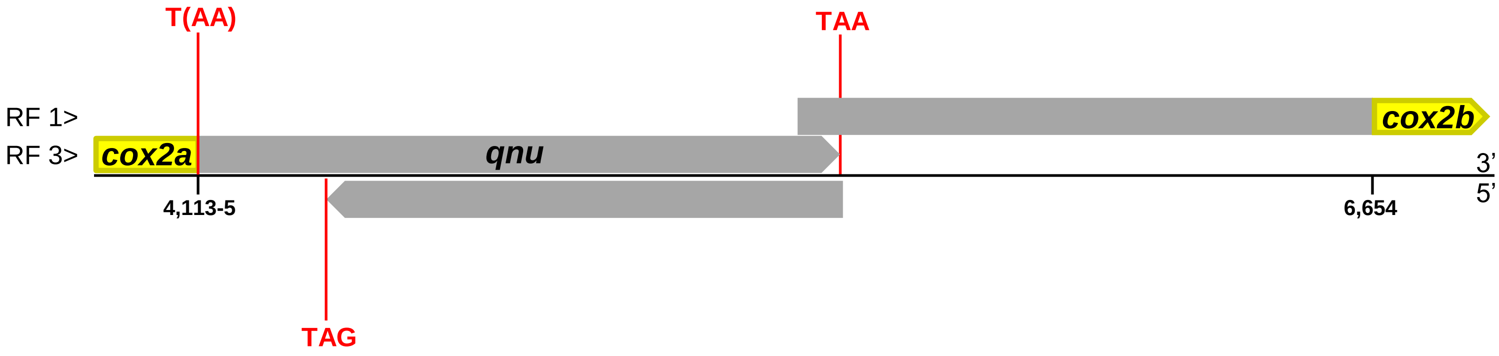

Supplementary Figure S9. Open reading frames (ORFs) greater than 0.5 kb, present in the *cox2*-splitting insert of *D. tejensis* sp. nov. The 3’-end of *cox2a* and the 5’-end of *cox2b* are highlighted in yellow. Positions of the stop codons are in red.

```
cox2a 4103 TAGATTTGCTTCAGAAAAAATAATAAACTTATCAAACAATAAATCTAAATCTAATTATGAAATATTAACATTT-AAAAATAA
      6320 ATGTTAGACAAGAAGGATTTACAAGACCTAATCGCCGAACTTATCCTTCTATTGTTATTACCCAACCTTCTTTCGAAAGTGA
             * *   *   *   *     *  * ** ***    **   ***    * *  **** *   *      ***  *** * *

TTTAAATTTTA----TACAAAACTTTGAACTATTAGAATATTTAAAAGGATTAATTTTATCCTTTATTAACAACTTAAATTTTTGAATTA---
AGAAGAATTAACACAAAATAATCTTTCAAATATTAGTATTAATGTTAGTGTTCCTACTCCCCCTAA--AACAACCTCCTTATCTAAAATAGTA
   * * ** *     *  ** **** ** ****** **   *   **  **  *  *  ** * *  ****** *   * * * ** **

------------AAAATTCAACTAACTTATTATTA---------------ATTCATTTTAATGGAGTAGGTACAAGTCTAATTAATGAAGAAT
AAAGAATTATTTAAGTGTGAACTATGTTATTATGACCCTCCTTCTTCACCATGCAATATAATTGACAGATT--AAATATTTTTAAAGATGAAA
            **   * *****  ******* *               ** ** * **** **     *  ** * *  **** ** ***

CTCCTTTCCATTTAGTTAACA--------------------------ACAATAAATGAATACATAGAAGAGAATCAGCAGAC 4391
CATATAACAATATAGATTACATAAAAGAAGAATCTGAAGAAAAAATTACTAAAAATGTAAAAGTAGAAGAAATTAAATACTT 6665 cox2b
*   *  * ** *** * ***                          ** * ***** * *  ******* * * *  *
```

Supplementary Figure S10. Pairwise sequence alignment in Lalign of the putative remnants of the directly oriented DNA sequence blocks bounding the region that separates *cox2a* and *cox2b* in *D. tejensis* sp. nov. Asterisks indicate nucleotide identities.

Supplementary Table S1. Detailed annotation of the mitogenomes of *D. tejensis* sp. nov. and *D. p. fossulana*. The *trn* genes, with the exception of *trnS1*s, *trnR* and *trnG*, were detected by tRNAscan. Intergenic regions larger than 25 bp, except for the CR and the *cox2*-splitting insert, are highlighted in green. Differences between the *trn* genes/pseudogenes of the two species are highlighted in yellow. Shaded coordinates refer to an assumption that there is no gene overlapping.

***Dielis tejensis* sp. nov.**								***Dielis p. fossulana***							
**Gene**	**mtDNA strand[a]**	**Location[b]**	**Size (bp)**	***trn* anticodon / loop replacement[c]**	**Start codon**	**Stop codon**	**Integenic region (bp)[d]**	**Gene**	**mtDNA strand[a]**	**Location[b]**	**Size (bp)**	***trn* anticodon / loop replacement[c]**	**Start codon**	**Stop codon**	**Integenic region (bp)[d]**
trnD	-	1-67	67	gtc			1	*trnD*	-	1-67	67	gtc			5
Invertion															
trnQ	+	69-136	68	ttg			-1	*trnQ*	+	73-140	68	ttg			-1
trnS1	+	136-196	61	tct/D-loop			5	*trnS1*	+	140-199	60	tct/D-loop			5
trnI	+	202-268	67	gat			-1	*trnI*	+	205-272	68	gat			-1
trnS1	+	268-333	66	act/D-loop			-3	*trnS1*	+	272-337	66	act/D-loop			-3
trnM	+	331-398	68	cat			2	*trnM*	+	335-402	68	cat			2
trnL2	+	401-464	64	taa			0	*trnL2*	+	405-467	63	taa			0
trnM	+	465-538	74	cat			6	*trnM*	+	468-548	81	cat			5
trnI	+	545-611	67	aat/p			0	*trnH*	+	554-619	66	atg/p			0
nad2	+	612-1658	1047		ATA	TAG	186 (O_R)	*nad2*	+	620-1654	1035		ATG	TAA	25
trnW	+	1845-1915	71	tca			-10	*trnW*	+	1680-1742	63	tca			-8
trnC	-	1906-1969	64	gca			8	*trnC*	-	1735-1798	64	gca			8
trnY	-	1978-2043	66	gta			4	*trnY*	-	1807-1872	66	gta			5
cox1	+	2048-3619	1572		ATG	TAA	58	*cox1*	+	1878-3449	1572		ATG	TAA	74
cox2a	+	3678-4113	436		ATT	Taa[e]	130	*cox2a*	+	3524-3869	346		ATG	Taa[e]	167
cox2-splitting insertion															
trnK	-	4244-4304	61	ttt/TV-loop/p			2352	*trnN*	+	4037-4093	57	gtt/TV-loop/p			2063
								trnL1	-	6157-6233	77	gag/p			614
End of the insertion															
cox2b	+	6655-6984	330		ATT	TAA	95	*cox2b*	+	6848-7150	303		ATA	TAA	97
trnK	+	7080-7152	73	ttt			257	*trnK*	+	7248-7319	72	ctt			0
trnK	+	7410-7478	69	ttt			0								
End of the inversion															
CR[f]		7479-7774					0	CR[f]		7320-7497					0
rrnS	+	7775-8573	799				1	*rrnS*	+	7498-8294	797				0
trnV	+	8575-8639	65	tac			-1	*trnV*	+	8295-8361	67	tac			0
rrnL	+	8639-9992	1354				1	*rrnL*	+	8362-9709	1348				0
trnL1	+	9994-10063	70	tag			0	*trnL1*	+	9710-9780	71	tag			0
nad1	+	10064-10993	922		ATG	TAA	-7	*nad1*	+	9781- 10710	922		ATG	TAA	-7
trnS2	-	10987-11053	67	tga			13	*trnS2*	-	10704-10769	66	tga			10
cob	-	11067-12248	1182		ATA	TAA	22	*cob*	-	10780-11967	1188		ATT	TAA	23
nad6	-	12271-12831	561		ATT	TAA	11	*nad6*	-	11991-12563	573		ATT	TAA	0
trnP	+	12843-12908	66	tgg			3	*trnP*	+	12564-12629	66	tgg			7
trnT	-	12912-12977	66	tgt			1	*trnT*	-	12637-12702	66	tgt			1
nad4L	+	12979-13260	282		ATT	TAG	5	*nad4L*	+	12704-12985	282		ATT	TAG	5
nad4	+	13266-14592	1327		ATA	Taa[e]	0	*nad4*	+	12991-14317	1327		ATA	Taa[e]	0
trnH	+	14593-14655	63	gtg			0	*trnH*	+	14318-14379	62	gtg			0
nad5	+	14656-16338	1683		ATC	TAA	-9	*nad5*	+	14380-16062	1683		ATT	TAA	-9
trnE	-	16330-16395	66	ttc			0	*trnE*	-	16054-16119	66	ttc			0
trnN	-	16396-16462	67	gtt			-3	*trnN*	-	16120-16186	67	gtt			-2
trnR	-	16460-16527	68	tcg			15	*trnR*	-	16185-16250	66	tcg			16
trnA	-	16543-16608	66	tgc			0	*trnA*	-	16267-16329	63	tgc			0
nad3	-	16609-16965	357		ATG	TAA	-1	*nad3*	-	16330-16683	354		ATG	TAG	0
trnG	-	16965-17036	72	acc			-1	*trnG*	-	16684-16752	69	acc			-2
trnF	+	17036-17102	67	gaa			2	*trnF*	+	16751-16817	67	gaa			2

cox3	-	17105-17890<br>17105-17902	786<br>798		ATA<br>ATG	TAA	4<br>-8	*cox3*	-	16820-17605<br>16820-17617	786<br>798		ATA<br>ATG	TAA	4<br>-8
atp6	-	17895-18563<br>17895-18575	669<br>681		ATA	TAA	5<br>-7	*atp6*	-	17610-18278	681		ATA	TAA	5
atp8	-	18569-18730	162		ATA	TAA	-1	*atp8*	-	18284-18445	162		ATG	TAA	0

[a]+ (forward) refers to the L strand (G + T = 49.97%); **-** (revers) refers to the H strand (G + T = 50.03%)

[b]Mitogenomic coordinates refer to the L strand sequences of *D. tejensis* sp. nov. (GenBank_MN990424) and *D. p. fossulana* (GenBank_KT740996) mtDNA.

[c]p, putative pseudogene

[d]The coding capacity of the *cox2*-splitting insert has yet to be determined

[e]Termination codon completed by polyadenylation

[f]The control region may extend upstream even to the 3’ end of *cox2b* and include one or both *trnK*(s)

Supplementary Table S2. Codon usage by mitochondrial PCGs in (**a**) *D. tejensis* sp. nov. (excluding *cox2*-splitting insert) and (**b**) *Drosophila littoralis*. Abbreviations: CU, number of individual codons; RSCU, relative synonymous codon usage. The initiation and termination codons were not included in the calculations. Similar to other Hymenoptera and insects in general, TTA (Leu), ATT (Ile), TTT (Phe) and ATA (Met) are the four most frequently used codons and respective amino acids.

a

Codon	Amino acid	CU	RSCU	Codon	Amino acid	CU	RSCU	Codon	Amino acid	CU	RSCU	Codon	Amino acid	CU	RSCU
AAA	K	157	1.85	GAA	E	79	1.88	CAA	Q	50	1.96	TAA	*	-	-
AAG	K	13	0.15	GAG	E	5	0.12	CAG	Q	1	0.04	TAG	*	-	-
AAC	N	27	0.26	GAC	D	9	0.24	CAC	H	12	0.34	TAC	Y	26	0.22
AAT	N	183	1.74	GAT	D	65	1.76	CAT	H	59	1.66	TAT	Y	208	1.78
AGA	S	95	2.15	GGA	G	92	2.22	CGA	R	26	2.42	TGA	W	83	1.87
AGG	S	4	0.09	GGG	G	18	0.43	CGG	R	3	0.28	TGG	W	6	0.13
AGC	S	8	0.18	GGC	G	12	0.29	CGC	R	4	0.37	TGC	C	4	0.18
AGT	S	40	0.90	GGT	G	44	1.06	CGT	R	10	0.93	TGT	C	41	1.82
ACA	T	60	1.63	GCA	A	33	1.50	CCA	P	27	0.96	TCA	S	79	1.79
ACG	T	3	0.08	GCG	A	0	0	CCG	P	1	0.04	TCG	S	9	0.20
ACC	T	4	0.11	GCC	A	6	0.27	CCC	P	10	0.36	TCC	S	5	0.11
ACT	T	80	2.18	GCT	A	49	2.23	CCT	P	74	2.64	TCT	S	114	2.58
ATA	M	326	1.91	GTA	V	106	2.29	CTA	L	39	0.46	TTA	L	393	4.65
ATG	M	16	0.09	GTG	V	2	0.04	CTG	L	2	0.02	TTG	L	24	0.28
ATC	I	28	0.13	GTC	V	2	0.04	CTC	L	1	0.01	TTC	F	16	0.09
ATT	I	388	1.87	GTT	V	75	1.62	CTT	L	48	0.57	TTT	F	340	1.91

b

Codon	Amino acid	CU	RSCU	Codon	Amino acid	CU	RSCU	Codon	Amino acid	CU	RSCU	Codon	Amino acid	CU	RSCU
AAA	K	74	1.76	GAA	E	70	1.82	CAA	Q	72	1.92	TAA	*	-	-
AAG	K	10	0.24	GAG	E	7	0.18	CAG	Q	3	0.08	TAG	*	-	-
AAC	N	34	0.33	GAC	D	15	0.45	CAC	H	25	0.65	TAC	Y	43	0.51
AAT	N	170	1.67	GAT	D	52	1.55	CAT	H	52	1.35	TAT	Y	124	1.49
AGA	S	68	1.65	GGA	G	105	1.87	CGA	R	35	2.378	TGA	W	89	1.76
AGG	S	1	0.02	GGG	G	52	0.92	CGG	R	4	0.27	TGG	W	12	0.24
AGC	S	9	0.22	GGC	G	5	0.09	CGC	R	1	0.07	TGC	C	2	0.09
AGT	S	22	0.53	GGT	G	63	1.12	CGT	R	19	1.29	TGT	C	41	1.91
ACA	T	87	1.86	GCA	A	47	1.09	CCA	P	33	1.00	TCA	S	102	2.48
ACG	T	3	0.06	GCG	A	12	0.28	CCG	P	9	0.27	TCG	S	9	0.22
ACC	T	21	0.45	GCC	A	22	0.51	CCC	P	17	0.52	TCC	S	13	0.32
ACT	T	76	1.63	GCT	A	92	2.13	CCT	P	73	2.21	TCT	S	105	2.55
ATA	M	192	1.80	GTA	V	79	1.56	CTA	L	33	0.33	TTA	L	476	4.71
ATG	M	21	0.20	GTG	V	11	0.22	CTG	L	3	0.03	TTG	L	50	0.50
ATC	I	40	0.23	GTC	V	10	0.20	CTC	L	3	0.03	TTC	F	27	0.16
ATT	I	312	1.77	GTT	V	102	2.02	CTT	L	41	0.41	TTT	F	303	1.84

Supplementary Table S3. List of amino acid residues predicted to interact at the COXIIA/COXIIB docking interface in *D. tejensis* sp. nov.

Residue	Molecule	COXIIA-COXIIB intermolecular contacts	Residue	Molecule	COXIIB-COXIIA intermolecular contacts
V 5	COXIIA	R 57, G 56, P 55	M 1	COXIIB	L 106, K 104
Y 7		R 57, G 56, L 58	R 19		N 108, D 127, T 113
Q 15		G 56	M 21		N 108, Q 109, G 107, W 110
V 18		G 56, L 36, P 55	M 23		Q 109
S 19		D 37, P 55	I 29		Q 109
P 20		D 37	R 30		W 110
N 21		I 39, V 38	V 31		Q 109
M 22		G 56, P 55, V 54	L 32		N 131, L 130, W 112
M 25		I 39, V 54	T 33		W 112
N 92		I 39, I 78	S 34		L 130, W 112
D 93		C 79, I 78	S 35		W 112
E 94		C 79	L 36		L 130, V 18
G 95		C 79, G 80	D 37		P 20, S 19, V 103, V 105
T 96		L 81	V 38		N 21
L 97		L 81	I 39		N 21, N 92, M25
P 99		L 81	F 42		G 107, W 112
V 103		D 37	V 54		M 22, M 25
K 104		M 1, M 86, S 84	P 55		M 22, S 19, V 18, V 5
V 105		D 37, M 86	G 56		Q 15, M 22, Y 7, V 18, V 5
L 106		M 1, M 86, P 87	R 57		Y 7, V 5
G 107		M 21, F 42, F 88	L 58		L 130, Y 7
N 108		R 19, M 21	I 78		N 92, D 93
Q 109		I 29, I 90, M 21, V 23, V 31	C 79		D 93, G 94, G 95
W 110		R 30, M 21, F 102	G 80		G 95
F 111		F 102	L 81		L 97, P 99, T 96
W 112		L 32, F 42, S 34, S 35, T 33	S 84		K 104
T 113		R 19	M 86		L 106, K 104, V 105
D 127		R 19	P 87		L 106
L 130		L 32,L 36, L 58, S 34	F 88		G 107
N 131		L 32	I 90		Q 109
			F102		F 111, W 110

Supplementary Table S4. Hymenoptera featuring enlarged intergenic regions and supernumerary *trn* genes in their mitogenomes. Capital letters in parentheses represent *trn* gene symbols; PCG, protein-coding gene; r, rearranged region (ribosomal or protein gene defining the region has been relocated); Σ, total length of the non-coding sequence interrupted by *trn* genes; n/a, sequence not available or not analysed. Regions of particular interest because of their large size and/or the presence of duplicated *trn* genes are highlighted in green.

**Superfamily**	**Family / subfamily / tribe**	**Species**	**Genbank accession**	**Species No (Fig.4)**	**mtDNA length (bp)**	**Intergenic regions**			
						**(*rrnS-I-Q-M-nad2*) (≥ 50 bp-large)**	***nad2-W-C-Y-cox1* (≥ 50 bp-large)**	***cox1-L2-cox2* (≥ 50 bp-large)**	***nad3-A-R-N-S1-E-F-nad5* & other regions (≥ 100 bp-large)**
"Symphyta"									
Pamphilioidea	Megalodontidae	*Megalodontes quinquecinctus*	MG923502	1	>17,033	≥2,111 bp (*rrnS-W*)	- (no *W*)	-	-
	Pamphiliidae	*Chinolyda flagellicornis*	MH577057	2	>16,523	≥1,532 bp (*rrnS-M*)	-	-	-
Xyeloidea	Xyelidae	*Xyela* sp.	MG923517	3	>21,161	≥6,112 bp (*rrnS-I*), 97 bp (*Q-nad2*)	-	-	-
Tenthredinoidea	Pergidae	*Perga condei*	AY787816	-	>13,416	n/a	n/a	-	-
	Argidae	*Arge* sp.	MN913350	4	15,675	254 bp (*rrnS-W*), 199 bp (*I-M*)	- (no *W*)	-	-
	Tenthredinidae	*Eutomostethus vegetus*	MT663219	5	>16,345	≥105 bp (*rrnS-M*), 680 bp (*Q-I*)	-	159 bp (*cox1-L2*)	139 bp (*S1-E*)
	Diprionidae	*Neodiprion sertifer*	MK994526	6	16,461	650 bp (*rrnS-M*)	-	-	225 bp (*S1-R*), 104 bp (*R-N*), 280 bp (*E-N*)
	Cimbicidae	*Labriocimbex sinicus*	MH136623	7	15,405	350 bp (*Q-Y*)	- (no *Y,C*)	-	-
Xiphydrioidea	Xiphydriidae	*Xiphydria* sp.	MH422969	8	>16,482	95 bp (*I-M*) & n/a?	- (no *Y,C*)	-	111 bp (*T-nad1*)
Siricoidea	Siricidae	*Tremex columba*	NC040123	9	>16,397	≥212 bp (*rrnS-C*), 149 bp (*Y-V*)	- (no *Y,C*)	-	255 bp (*nad4L-T*), 333 bp (*T-P*), 266 bp (*P-nad6*)
Cephoidea	Cephidae	*Hartigia linearis*	KX907843	10	>20,116	223 bp (*M-D*), ≥3,731 bp (*D-Q*), 58 bp (*Q-I*)	-	-	-
		Calameuta liliformis	KT260167	11	20,055	4,469 bp (*M-Q*), 66 bp (*Q-I*), 68 bp (*I-nad2*)	-	-	-
		Calameuta idolon	KT260168	12	19,746	4,189 bp (*M-Q*), 63 bp (*Q-I*), 71 bp (*I-nad2*)	-	-	-
		Trachelus iudaicus	NC032071	13	20,370	145 bp (*rrnS-M*), 4,753 bp (*M-Q*), 59 bp (*Q-I*), 55 bp (*I-nad2*)	70 bp (*C-Y*), 69 bp (*Y-cox1*)	-	
		Trachelus tabidus	KX257358	14	18,539	3,094 bp (*M-Q*), 87 bp (*I-nad2*)	-	-	-
		Cephus cinctus	FJ478173	15	19,339	3,873 bp (*M-Q*), 60 bp (*I-nad2*)	-	-	-
Orussoidea	Orussidae	*Orussus occidentalis*	FJ478174	16	15,947	1,063 bp (*rrnS-Q*)	-	-	-
Apocrita / Proctotrupomorpha									
Cynipoidea	Ibaliidae	*Ibalia leucospoides*	KJ814197	17	17,212	r: (*W*-3x*M*-*L1*: Σ77 bp): 54 bp (*M-L1*) (*nad2* is inverted)	- (no *W,C*; *nad2* is inverted)	- (no *L2*)	465 bp (*E-rrnL*), 240 bp (*nad5-C*), 848 bp (*C-S2*), 256 bp (*S2-nad1*)
	Figitidae / Charipinae	*Alloxysta* sp.	MG923482	18	>15,882	r: ≥471 bp (*rrnS-S2*)	- (no *W,C*; *nad2* is inverted)	- (no *L2*)	-
	Figitidae / Eucoilinae	*Gastraspis* sp.	MG923497	19	>16,486	r: - (*nad2* is inverted)	- (no *W,C*; *nad2* is inverted)	- (no *L2*)	173 bp (*nad5-S2*), 161 bp (*A-F*), 327 bp (*N-S1*)
		Leptopilina boulardi	KU665622	20	15,417	441 bp (*C-rrnS*; *rrnS* is inverted), 247 bp (*E-nad2*; *nad2* is inverted)	57 bp (*nad2-Y*; *no W,C*; *nad2* is inverted)	- (*no L2*)	n/a
	Cynipidae / Cynipinae / Cynipini	*Trichagalma acutissimae*	MN928529	21	16,078	102 bp (*rrnS-C*)	- (no *W,C*; *nad2* is inverted)	- (no *L2*)	-
	Cynipidae / Cynipinae / Synergini	*Synergus* sp.	MG923514	22	>15,584	r: ≥354 bp (*C-S2*)	- (no *W,C*; *nad2* is inverted)	- (no *L2*)	-

Platygastroidea	Scelionidae / Scelioninae	*Habroteleia persimilis*	MG923508	23	>17,186	≥2,332 bp (*V-Q*)	-	-	-
		Idris sp.	KF696670	24	15,137	414 bp (*M-Q*)	- (no *Y*,*C*)	-	-
		Scelio sp.	MG923509	25	>16,851	≥2,094 bp (*F-V*)	- (no *Y*,*C*)	-	-
	Scelionidae / Telenominae	*Telenomus* sp.	MF776884	26	>17,023	999 bp (*V-C*), 73 bp (*C-Y*), ≥974 bp(*Y-Q*), 123 bp (*A-nad2*)	- (no *Y*,*C*)	-	60 bp (*nad6-cob*)
	Platygastridae	*Platygaster* sp.	MG923510	27	>16,605	≥1,962 bp (*M-Q*)	-	-	-
Proctotrupoidea	Monomachidae	*Monomachus antipodalis*	KM104168	28	>14,066	n/a	- (no *Y*)	- (*-S2-Y-V-L2-*)	298 bp (*cob-nad1*)
	Heloridae	*Helorus* sp.	MG923498	29	>16,946	n/a	100 bp (*Q-cox1*) (no *Y*,*C*)	-	-
	Vanhorniidae	*Vanhornia eucnemidarum*	DQ302100	30	16,574	62 bp (*L1-nad2*) & n/a (?)	-	107 bp (*cox1-cox2*) (*no L2*)	663 bp (*L2-K*), 159 bp (*D-atp8*), 71 bp (*cox3-nad3*), (*nad3*-[*S1-N-A*]$_5$*S1*): Σ247 bp, 60 bp (*S1-E*), 52 bp (*E-F*), 228 bp (*nad6-cob*)
	Pelecinidae	*Pelecinus polyturator*	NC026865	31	14,896	192 bp (*rrnS-Q*)	-	-	-
	Proctotrupidae	*Exallonyx* sp.	MG923495	32	>17,696	≥2,524 bp (*rrnS-I*)	-	70 (*L2-cox2*)	-
"Diaprioidea"	Diapriidae	Diapriidae sp.	MG923491	33	>17,411	≥2,275 bp (*rrnS-M*)	- (no *Y*)	-	152 bp (*cob-S2*)
	Diapriidae / Diapriinae	*Trichopria drosophilae*	NC048491	34	16,375	1,919 bp (*V-Q*)	- (no *Y*)	- (*no L2*)	-
	Diapriidae / Ismarinae	*Ismarus* sp.	MG923501	35	>16,969	≥2,082 bp (*V-M*)	- (no *Y*)	- (*no L2*)	64 bp (*cox3-nad3*), 72 bp (*L1-G*)
Chalcidoidea	Agaonidae	*Eupristina konigsbergeri*	MT947597	36	>15,400	r: - & n/a	r: -	-	-
	Mymaridae	*Gonatocerus* sp.	MF776883	37	15,554	509 bp (*M-I*)	109 bp (*C-Y*)	-	-
	Trichogrammatidae	*Trichogramma dendrolimi*	KU836507	38	16,878	1,320 bp (*M-W*) (*nad2* is inverted)	120 bp (*Q-Y*; no *W*,*C*), 87 bp (*Y-cox1*) (*nad2*, *cox1* is inverted)	r: -	-
	Chalcididae / Chalcidinae / Brachymerini	*Brachymeria* sp.	MG923487	39	>15,092	n/a, & (*-M-M-V*): 308 bp (*M-M*), 124 bp (*M-V*) (*nad2* is inverted; *rrnS* is missing)	r: -	-	-
	Eurytomidae / Eurytominae	*Eurytoma* sp.	MG923494	40	>17,267	≥2,267 bp (*M-Y*) (*nad2* is inverted)	r: 113 bp (*W-S1*) (*nad2* inverted)	-	-
	Encyrtidae / Encyrtinae	*Diaphorencyrtus aligarhensis*	NC046058	41	≥16,264	≥291 bp (*M-I*)	r: 53 bp (*W-S*), 51 bp (*C-Q*)	r: -	325 bp (*N-R*), 194 bp (*F-E*), 448 bp (*E-nad5*)
	Encyrtidae / Tetracneminae	*Aenasius arizonensis*	NC045852	42	15,373	397 bp (*M-I*)	r: -	-	-
	Aphelinidae / Coccophaginae	*Encarsia formosa*	MG813797	43	>17,736	69 bp (*rrnS-M*), ≥3,042 bp (*M-I*)	r: -	-	-
	Eupelmidae / Eupelminae	*Eupelmus* sp.	MG923493	44	>17,037	≥2,323 bp (*M-I*)	r: -	-	-
	Eulophidae	*Necremnus tutae*	MT916846	45	15,252	235 bp (*M-W*) (*nad2* is inverted)	r: - (*nad2* inverted)	-	-
	Pteromalidae / Pteromalinae	*Pteromalus puparum*	NC039656	46	18,217	(*M-I-I-nad2*): 3,309 bp (*I-I*)	r: -	-	-
	Pteromalidae / Sycoryctinae	*Philotrypesis pilosa*	JF808723	47	>15,122	r: ≥1,670 bp (*S2-I*), 62 bp (*I-nad2*) (*nad2* is inverted)	r: 67 bp (*Y-C*) (*nad2* inverted)	69 bp (*L2-cox2*)	-
	Torymidae / Toryminae	*Torymus* sp.	MG923516	48	>16,826	≥1,819 bp (*M-S2*) (*nad2* is inverted)	r: 72 bp (*nad2-Y*) (*nad2* is inverted)	-	120 bp (*cob-nad1*)
Apocrita / "Evaniomorpha" / Ceraphronoidea									
Ceraphronoidea	Ceraphronidae	Ceraphronidae sp.	MG923488	49	>15,560	r: ≥1,299 bp (*M-W*)	r: - (*nad2* is inverted)	-	-
		Ceraphron sp.	KJ570858	50	14,947	r: 692 bp (*M-W*)	r: - (*nad2* is inverted)	-	-
	Megaspilidae	*Conostigmus* sp.	KF015227	51	16,315	r: 1,447 bp (*rrnS-G*)	r: -	r: -	-
		Dendrocerus sp.	MG923490	52	>21,487	r: ≥5,734 bp (*rrnS-G*)	r: -	r: 104 bp (*cox1-L2*)	612 bp (t_*cox2-K*)
Apocrita / Ichneumonomorpha									

Ichneumonoidea	Ichneumonidae / Ophioninae	*Enicospilus* sp.	FJ478177	53	>15,300	≥325 bp (*rrnS-H*)	-	- (no *L2*)	133 bp (*nad1-L1*)
	Ichneumonidae / Metopiinae	*Hypsicera* sp.	MG923500	54	>17,017	n/a	-	-	n/a
	Ichneumonidae / Campopleginae / *Dusona* group	*Diadegma fenestrale*	MN599978	55	20,849	5,587 bp (*rrnS-L2*), 103 bp (*Q-nad2*)	-	152 bp (no *L2*)	101 bp (*P-T*)
		Diadegma semiclausum	NC012708	56	18,728	2,161 bp (*rrnS-L2*), 57 bp (*Q-nad2*)	-	1,515 bp (no *L2*)	-
		Hyposoter sp.	MG923499	57	>18,893	≥3,849 bp (*rrnS-L2*)	-	- (no *L2*)	-
	Ichneumonidae / Pimplinae	*Pimpla luctuosa*	MG923506	58	>16,926	≥2,023 bp (*rrnS-M*)	-	-	-
	Ichneumonidae / Ichneumoninae	*Amblyjoppa* sp.	MG923483	59	>17,110	≥2,174 bp (*rrnS-M*)	-	-	-
	Braconidae / Alysiinae	*Asobara japonica*	MN882556	60	15,519	696 bp (*I-Q*)	-	-	-
	Braconidae / Opiinae	*Diachasmimorpha longicaudata*	GU097655	61	>13,850	>715 bp (*M-?*)	n/a	-	-
	Braconidae / Doryctinae	*Spathius agryli*	FJ387020	62	15,425	578 bp (*M-Q*)	-	-	64 bp (*nad5-nad4*)
	Braconidae / Microgastrinae	*Cotesia vestalis*	FJ154897	63	15,543	571 bp (*M-Q*)	-	-	-
	Braconidae / Cardiochilinae	*Cardiochiles fuscipennis*	KF385870	64	>14,390	≥277 bp (*rrnS-nad2*)	-	-	-
	Braconidae / Euphorinae	*Dinocampus coccinellae*	MG253265	65	>15,684	≥1,012 bp (*M-G*)	-	-	105 bp (*T-P*)
	Braconidae / Macrocentrinae	*Macrocentrus camphoraphilus*	GU097656	66	>15,801	905 bp (*I-S2*), ≥539 bp (*S2-C*)	n/a & -	-	-
	Braconidae / Zeleinae	*Zele chlorophthalmus*	NC039181	67	16,661	803 bp (*M-Q*)	- (no *C*)	-	(*S2-L2-cob*): 237 bp (*S2-L2*), 550 bp (*L2-cob*)
Apocrita / “Evaniomorpha”									
Stephanoidea	Stephanidae	*Foenatopus ruficollis*	KR270642	68	>14,434	- & n/a	- (no *Y*)	-	127 bp (*R-nad5*) & n/a
“Evanioidea”	Evaniidae	*Evania appendigaster*	FJ593187	69	17,817	2,325 bp (*W-C*)	- (no *W*,*C*)	-	534 bp (*K-D*), 244 bp (*atp8-atp6*)
		Prosevania sp.	MG923512	70	>16,466	≥1,854 bp (*W-M*)	- (no *C*,*W*)	-	238 bp (*nad5-H*)
		Parevania sp.	MG923505	71	>16,074	≥1,431 bp (*W-C*)	- (no *C*,*W*)	-	-
	Gasteruptiidae	*Gasteruption* sp.	KJ619460	72	17,884	r: -	1,033 bp (*cox1-L2*), 803 bp (*L2-W*) (*nad2-trns-cox1* is inverted)	r: 94 bp (*Q-cox2*)	188 bp (*C-Y*)
		Gasteruption parvicollarium	KR270643	73	17,009	r: -	924 bp (*cox1-L2*) (*nad2-trns-cox1* is inverted)	r: -	241 bp (*C-Y*)
	Aulacidae	*Aulacus sinensis*	MG923485	74	16,953	r: 113 bp (*rrnL-M*)	-	-	233 bp (*A-G*), 542 bp (*G-R*), 876 bp (*S2-nad1*)
Megalyroidea	Megalyridae	*Megalyra* sp.	KJ577600	75	18,996	r: -	r: 62 bp (*nad2-V*)	-	3,871 bp (*L1-Q*)
Trigonaloidea	Trigonalidae	*Orthogonalys pulchella*	NC025289	76	17,277	88 bp (*Q-nad2*)	r: (*nad2-E-E-C*): 244 bp (*E-E*), 1,744 bp (*E-C*)	r: -	-
		Taeniogonalos tailorina	NC027830	77	15,927	-	r: -	r: -	D-loop: 921 bp (*nad3-D*)
Apocrita / Aculeata									
Chrysidoidea *s. stricto*	Embolemidae	*Embolemus* sp.	MH748652*	78	14,930	566 bp (*I-M*)	-	-	-
	Bethylidae	*Cephalonomia gallicola*	FJ823227	79	>16,720	60 bp (*Y-Q*)	- (no *trns*)	-	-

	Chrysididae / Cleptinae	*Cleptes metallicorpus*	MG923489	80	>15,813	-	1,007 bp (*K-cox1*)	- (*D,Q* but no *L2*)	-
	Chrysididae / Chrysidinae	*Chrysis* sp.	MH748672*	81	15,455	r: 500 bp (*L2-M*) (*rrn*s are inverted)	r: -	51 bp (no *L2*)	-
Chrysidoidea *s. lato*	Dryinidae / Dryininae	*Dryinus* sp.	MG923492	82	>17,069	≥1,147 bp (*V-Q*), 60 bp (*Q-M*)	-	-	118 bp (*nad4L-T*), 289 bp (*S2-nad1*)
	Dryinidae / Gonatopodinae	*Haplogonatopus apicalis*	MH748653*	83	15,916	r: 1,011 bp (*P-G*);<br>r: 73 bp (*Q-nad3*) (*nad2-trns-cox3* is inverted)	-	-	-
Vespoidea	Vespidae / Stenogastrinae	*Eustenogaster scitula*	NC044146	84	17,867	3,016 bp (*V-I*), 118 bp (*I-nad2*)	- (*W,Q,M,H,C,Y*)	-	-
	Vespidae / Eumeninae	*Abispa ephippium*	EU302588	85	16,953	308 bp (*rrnS-L1*), (*L1-M-Q-M-I*): Σ17 bp, 71 bp (*I-nad2*)	172 bp (*nad2-W*)	(4 x *L2*): Σ117 bp	750 bp (*S2-nad1*)
		Ancistrocerus sp.	MH748667*	86	18,005	239 bp (*rrnS-I*), (*I-M-M-Q-nad2*): 2,597 bp (*M-M*)	-	-	-
		Anterhynchium abdominale	MK051029	87	>16,488	≥1,406 bp (*rrnS-M*)	52 bp (*nad2-W*)	-	153 bp (*S1-E*)
		Orancistrocerus aterrimus	NC039949	88	17,972	1,078 bp (*rrnS-I*), (*I-M-Q-M-nad2*): 1,946 bp (*M-Q*)	-	-	-
		Pararrhynchium sp.	MH748657*	89	16,727	1,342 bp (*rrnS-I*), (*I-M-M-nad2*): 400 bp (*M-M*)	-	-	n/a
		Rhynchium aff. brunneum	NC048884	90	23,251	(*V-M-I-M-L1*): 1,273 bp (*V-M*), 56 bp (*M-I*), 5,806 bp (*I-M*), 73 bp (*M-L1*), 87 bp (*Q-nad2*)	-	-	107 bp (*A-R*), 130 bp (*nad4L-T*), 515 bp (*S2-nad1*)
	Vespidae / Polistinae / Polistini	*Polistes jokahamae*	KR052468	91	>16,616	≥1,096 bp (*rrnS-M*)	- (no *Y*)	-	123 bp (*D-K*), 200 bp (*K-atp8*), 138 bp (*G-nad3*), 201 bp (*nad6-cytB*)
	Vespidae / Polistinae / Ropalidiini	*Parapolybia crocea*	NC036343	92	16,619	1,316 bp (*rrnS-Y*), 135 bp (*M-nad2*)	- (no *Y*)	-	108 bp (*L1-nad1*)
		Ropalidia fasciata	MK034145*	93	18,605	1,508 bp (*rrnS-Y*), (*Y-I-Q-M-I-Q-M-nad2*): 1,068 bp (*Y-I*), 581 bp (*M-I*), 47 bp (*M-nad2*)	- (no *Y*)	-	-
		Ropalidia variegata	MK034148*	94	15,897	839 bp (*rrnS-I*), 60 bp (*M-nad2*)	- (no *Y*)	-	-
	Vespidae / Vespinae	*Vespa affinis*	NC039134	95	19,109	172 bp (*rrnS-Y*), 279 bp (*Y-I*), 333 bp (*I-M*), 315 bp (*Q-nad2*)	210 bp (*C-cox1*) (no *Y*)	-	121 bp (*K-D*), 245 bp (*R-N*), 618 bp (*N-E*), 107 bp (*S1-F*), 421 bp (*P-nad6*), 556 bp (*S2-L1*), 455 bp (*L1-nad1*)
		Vespa basalis	MK440075	96	16,735	808 bp (*rrnS-Y*), 79 bp (*Q-nad2*)	- (no *Y*)	-	103 bp (*K-D*), 127 bp (*L1-nad1*)
		Vespa bicolor	KJ735511	97	>16,937	≥898 bp (*rrnS-M*)	252 bp (*C-cox1*) (no *Y*)	-	207 bp (*K-D*), 171 bp (*L1-nad1*)
		Vespa crabro	MT862429	98	>14,981	1,135 bp (*rrnS-Y*), 150 bp (*I-nad2*)	52 bp (*nad2-W*) (no *Y*)	-	n/a
		Vespa mandarinia	NC050197	99	20,831	5,030 bp (*I-Y*), 96 bp (*Y-M*)	- (no *Y*)	-	131 bp (*H-nad4*), 118 bp (*L-nad1*)
		Vespa simillima	NC046020	100	18,340	1,600 bp (*rrnS-Y*)	146 bp (*C-cox1*) (no *Y*)	-	189 bp (*K-D*), 106 bp (*R-N*), 390 bp (*N-E*), 490 bp (*S1-F*), 186 bp (*L1-nad1*)
		Vespa magnifica	MT137097	101	16,730	1,563 bp (*V-Y*)	- (no *Y*)	-	145 bp (*R-E*), 182 bp (*S1-F*), 210 bp (*H-nad4*), 125 bp (*L1-nad1*)
		Vespa velutina auraria	MT137096	102	18,613	2,230 bp (*rrnS-Y*)	229 bp (*C-cox1*)	-	135 bp (*K-D*), 103 bp (*R-N*), 209 bp (*N-E*), 164 bp (*S1-F*), 180 bp (*L1-nad1*)
		Vespula flaviceps	NC045215	103	17,489	1,302 bp (*rrnS-Y*), 125 bp (*Q-nad2*)	- (no *Y*)	-	150 bp (*R-N*), 144 bp (*P-nad6*), 563 bp (*L1-*

									nad1)
		Vespula vulgaris	MK737083	104	>17,996	n/a	- (no *Y*)	-	316 bp (*L-nad1*)
		Dolichovespula panda	NC036067	105	>17,137	≥586 bp (*rrnS-Y*), 399 bp (*I-M*), 216 bp (*M-Q*)	- (no *Y*)	-	133 bp (*K-D*), 117 bp (*N-F*), 404 bp (*L1-nad1*)
	Vespidae *incertae sedis*	*Antodynerus aff. limbatus*	NC048883	106	17,975	876 bp (*rrnS-I*), (*I-M-Q-M-nad2*): 1,937 bp (*M-Q*)	-	-	-
		Allorhynchium sp.	NC048881	107	19,646	1,898 bp (*rrnS-I*), (*I-M-Q-M-nad2*): 2,738 bp (*M-Q*), 54 bp (*Q-M*)	-	-	-
Tiphioidea	Tiphiidae	*Tiphia* sp.	MH748663*	108	15,868	1,020 bp (*rrnS-W*) (*nad2* is inverted)	74 bp (no *trns*; *nad2-trns* is inverted)	-	-
		Tiphiidae sp. 2	MH748664*	109	15,609	702 bp (*rrnS-Y*), 197 bp (*I-Q*), 53 bp (*W-nad2*)	190 bp (no *trns*; *nad2-trns* is inverted)	-	-
Pompiloidea	Pompilidae	*Agenioideus* sp.	KX584356	110	16,596	66 bp (*Q-nad2*)	-	-	351 bp (*cox3-G*), 1,240 bp (*A-L1*)
		Auplopus sp.	KX584357	111	>16,746	57 bp (*Q-nad2*)	-	-	304 bp (*nad6-cob*), ≥1,754 bp (*nad1-A*)
	Mutillidae	*Wallacidia oculata*	FJ611801	112	18,442	1,874 bp (*rrnS-M*)	246 bp (*C-cox1*)	81 bp (*cox1-L2*)	129 bp (*atp6-cox3*), (*rrnL-rrnS*): 1,941 bp (*V-I*)
		Mutillidae sp. 2	MH748655*	113	16,389	1,335 bp (*rrnS-M*)	204 bp (*C-cox1*)	95 bp (*cox1-L2*)	(*rrnL-rrnS*): 243 bp (*V-I*)
Scolioidea	Scoliidae / Scoliinae / Campsomerini	*Dielis plumipes fossulana*	KT740996	114	18,445	r: 179 bp (*rrnS-K*), 97 bp (*K-cox2b*);<br>r: 65 bp (*Q-I*), (*S1-M-L2-M*-Hp-*nad2*): Σ2 bp, 71 bp (*M-nad2*) (*nad2-cox2b* is inverted)	- (25 bp: *nad2-W*)	74 bp (no *L2*)	2,978 bp (*cox2a-cox2b*)
		Dielis tejensis sp. nov.	MN990424	115	18,730	r: 296 bp (*rrnS-K*), 257 bp (*K-K*), 95 bp (*K-cox2b*);<br>r: 64 bp (*Q-I*), (*S1-M-L2-M*-Ip-*nad2*): Σ2 bp, 73 bp (*M-nad2*) (*nad2-cox2b* is inverted)	186 bp (*nad2-W*)	58 bp (no *L2*)	2,541 bp (*cox2a-cox2b*)
		Megacampsomeris prismatica	MH748671*	116	16,629	r: 1,254 bp (*rrnS-cox2a*);<br>r: 64 bp (*Q-I*), (*S1-M-L2-M-N*): Σ17 bp	431 bp (*nad2-W*)	34 bp (no *L2*)	n/a
	Scoliidae / Scoliinae / Scoliini	*Scolia bicincta*	KT276222	117	n/a	n/a & r: (*K-M-I-M-L2*): Σ20 bp	-	138 bp (no *L2*)	n/a
	Scoliidae	Scoliidae sp. 1	MH748661*	118	15,926	r: 151 bp (*rrnS-K*), 332 bp (*K-K*);<br>r: (*Q-M-I-M-L2*): Σ0 bp, 66 bp (*Q-M*) (*nad2-cox2* is inverted)	-	84 bp (no *L2*)	119 bp (*nad3-A*)
		Scoliidae sp. 2	MH748660*	119	16,325	r: 670 bp (*rrnS-K*);<br>r: (*S1-M-I-M-L2*): Σ32 bp, 43 bp (*nad5-E*) (*nad2-cox2* is inverted)	-	60 bp (*no L2*)	121 bp (*nad4-nad4L*)
Formicoidea	Formicidae / Ponerinae	*Ectomomyrmex javanus*	NC042678	120	15,512	822 bp (*Q-nad2*)	-	-	-
	Formicidae / Formicinae	*Acropyga fuhrmanni*	MH158405	121	15,568	406 bp (*rrnS-M*), 54 bp (*I-Q*), 55 bp (*Q-nad2*)	-	-	-
		Acropyga kinomurai	NC046423	122	18,287	346 bp (*rrnS-M*), 1,105 bp (*I-Q*), 107 bp (*Q-nad2*)	57 bp (*C-Y*), 64 bp (*Y-cox1*)	-	119 bp (*A-R*), 121 bp (*R-N*), 104 bp (*N-S1*), 178 bp (*S1-E*), 148 bp (*nad4-nad4L*), 200 bp (*P-nad6*)
		Acropyga myops	NC046424	123	16,659	589 bp (*rrnS-M*), 51 bp (*I-Q*), 133 bp (*Q-nad2*)	68 bp (*W-C*), 102 bp (*C-Y*), 85 bp (*Y-cox1*)	105 bp (*cox1-L2*)	109 bp (*cox3-G*)
		Acropyga pallida	NC046425	124	16,886	613 bp (*rrnS-M*), 112 bp (*Q-nad2*)	167 bp (*C-Y*)	84 bp (*cox1-L2*)	101 bp (*cox2-K*), 160 bp (*cox3-G*)
		Acropyga sauteri	NC046398	125	16,976	691 bp (*rrnS-M*), 49 bp (*I-Q*), 111 bp (*Q-nad2*)	73 bp (*C-Y*), 125 bp (*Y-cox1*)	57 bp (*cox1-L2*)	99 bp (*nad4-nad4L*)
		Anoplolepis gracilipes	NC039576	126	16,943	893 bp (*rrnS-M*), 50 bp (*M-I*), 54 bp (*Q-nad2*)	60 bp (*Y-cox1*)	-	164 bp (*S2-nad1*)
		Brachymyrmex patagonicus	MG253259	127	>17,107	1,023 bp (*rrnS-I*), 147 bp (*Q-nad2*)	n/a	-	120 bp (*cox3-nad3*), 984 bp (*A-nad5*), 106

									bp (T-P)
		Nylanderia flavipes	MN654113	128	16,687	960 bp (rrnS-I), 95 bp (Q-nad2)	91 bp (W-C)	50 bp (cox1-L2)	103 bp (cob-S2)
	Formicidae / Myrmicinae	*Aphaenogaster famelica*	MK801109	129	19,464	632 bp (rrnS-V), 1,020 bp (V-M), 1,104 bp (I-Q), 89 bp (Q-nad2)	64 bp (W-C), 151 bp (Y-cox1)	-	365 bp (E-F), 157 bp (T-P), 247 bp (cob-S2)
		Atta texana	MF417380	130	19,709	806 bp (rrnS-V), 104 bp (V-M), 248 bp (M-I), 53 bp (I-Q), 197 bp (Q-nad2)	173 bp (Y-coxI)	163 bp (cox1-L2)	175 bp (cox2-K), 121 bp (D-atp8), 171 bp (cox3-G), 113 bp (nad3-A), 329 bp (R-N), 162 bp (nad4-nad4L), 407 bp (nad6-cob), 139 bp (cob-S), 451 bp (S2-nad1), 266 bp (nad1-L)
		Pheidole obscurithorax	MG253277	131	>18,704	1,990 bp (rrnS-M), ≥1,409 (Q-nad2)	-	-	586 bp (F-nad5), rrnL is dup/inv
		Solenopsis geminata	HQ215537	132	15,552	376 bp (N-V), 58 bp (V-M), 78 bp (Q-nad2)	-	-	-
	Formicidae / Dolichoderinae	*Dolichoderus quadripunctatus*	NC049088	133	16,017	561 bp (rrnS-M), 96 bp (Q-nad2)	-	-	224 bp (H-nad4), 124 bp (cob-S2)
	Formicidae / Pseudomyrmecinae	*Pseudomyrmex feralis*	BK010379	134	18,835	566 bp (rrnS-M), 161 bp (I-Q), 69 bp (Q-nad2)	229 bp (W-C), 137 bp (C-Y), 216 bp (Y-cox1)	-	166 bp (cox2-K), 426 bp (atp8-atp6), 175 bp (cox3-G), 302 bp (nad3-A), 119 bp (N-S1), 225 bp (S1-E), 105 bp (E-F), 294 bp (nad4L-T), 361 bp (T-P), 139 bp (S2-nad1)
Apoidea / "Spheciformes"	Ampulicidae	*Ampulex compressa*	KX494110	135	>15,501	>89 bp (rrnS-M), 99 bp (M-Q), 119 bp (Q-I)	-	-	-
	Sphecidae	*Sceliphron madraspatanum*	KX494105	136	>16,551	>1,178 bp (Y-A)	- (no trns)	-	118 bp (S2-C), 100 bp (nad6-M)
	Crabronidae / Philanthinae	*Philanthus triangulum*	NC017007	137	16,029	1,039 bp (I-M), 60 bp (Q-nad2)	-	-	-
		Cerceris sp.	KX494109	138	>16,158	≥722 bp (rrnS-I), 63 bp (Q-nad2)	-	-	-
Apoidea / Anthophila	Melittidae	*Dasypoda hirtipes*	MT985326*	139	18,594	3,810 bp (rrnS-M)	-	-	-
		Melitta schultzei	MT985327*	140	20,230	r: 2,981 bp (nad3-rrnS) (nad2-nad3 is inverted)	-	219 bp (L2-A), 174 bp (A-N), 74 bp (E-cox2)	1,202 bp (S1-D)
		Rediviva intermixta	NC030284	141	16,875	1,187 bp (rrnS-M), 78 bp (Q-nad2)	181 bp (C-Y), 310 bp (W-cox1)	-	103 bp (P-nad6)
		Samba griseonigra	MT985328*	142	16,978	1,987 bp (rrnS-M)	-	-	88 bp (nad6-cob)
	Andrenidae	*Andrena camellia*	KX241615	143	15,065	396 bp (rrnS-I), 73 bp (M-Q)	-	-	-
		Andrena cineraria	KT164628	144	>17,069	≥2,325 bp (rrnS-I), 68 bp (M-Q),	-	-	-
	Colletidae / Colletinae	*Colletes gigas*	NC026218	145	15,885	539 bp (V-M)	63 bp (Y-W)	86 bp (cox1-L2)	147 bp (F-nad5)
	Colletidae / Hylaeninae	*Hylaeus dilatatus*	NC026468	146	15,475	452 bp (V-I), 178 bp (I-M), 74 bp (Q-nad2)	-	-	-
	Halictidae	*Nomia chalybeata*	NC051484	147	16,692	1,044 bp (rrnS-M), 219 bp (I-Q), 105 bp (Q-nad2)	-	-	n/a
	Megachilidae / Megachilinae	*Euaspis polynesia*	MT909816	148	17,682	2,128 bp (rrnS-A), 105 bp (I-Q), 173 bp (Q-nad2)	-	-	n/a
		Osmia excavata	KX494106	149	>17,536	>2,139 bp (rrnS-C), 488 bp (C-M), 110 bp (A-Q)	-	-	-
	Apidae / Nomadinae	*Nomada goodeniana*	KT164660	150	>15,201	>405 bp (rrnS-A), 139 bp (Q-nad2)	-	-	-
	Apidae / Apinae / Anthophorini	*Amegilla calceifera*	MW281320	151	17,728	2,552 bp (Q-A), 65 bp (A-nad2)	77 bp (Y-cox1)	-	224 bp (I-G)
		Habropoda radoszkowskii	MT436266	152	18,497	2,882 bp (rrnS-A), 62 bp (Q-nad2)	-	-	275 bp (T-P), 248 bp (nad6-cob)
	Apidae / Apinae / Corbiculata / Apini	*Apis andreniformis*	KF736157	153	>17,529	>1,123 bp (rrnS-S1), (M-4xL1-Q-L1-A): 68 bp (L1-L1)x3=204 bp, 68 bp (L1-Q), 68 bp (Q-L1), 69 bp (L1-A), 55 bp (I-nad2)	117 bp (nad2-C), 90 bp (Y-W)	-	118 bp (atp6-cox3), 225 bp (P-nad6)

		Apis cerana	KX908206	154	15,904	567 bp (*rrnS-S1*), 227 bp (*M-Q*)	-	89 bp (*L2-cox2*)	-
		Apis dorsata	NC037709	155	15,892	714 bp (*rrnS-S1*), 82 bp (*E-M*)	81 bp (*Y-W*)	25 bp (*L2-cox2*)	-
		Apis florea	NC021401	156	17,694	1,987 bp (*rrnS-E*), (*E-3xS1-M*): 108 bp (*S1-S1*)x2=216, 110 bp (*S1-M*), 58 bp (*Q-A*)	-	35 bp (*L2-cox2*)	-
		Apis koschevnikovi	KY348372	157	>16,050	898 bp (*rrnS-S1*), (*E-M-M-Q*): Σ46 bp	-	90 bp (*L2-cox2*)	-
		Apis laboriosa	KX908208	158	15,621	470 bp (*rrnS-S1*)	-	28 bp (*L2-cox2*)	n/a
		Apis mellifera anatoliaca	MT188686	159	16,256	756 bp (*rrnS-E*), 49 bp (*E-S1*)	62 bp (*Y-W*)	193 bp (*L-cox2*)	n/a
		Apis mellifera ligustica	MH341407	160	16,449	902 bp (*rrnS-E*), 49 bp (*E-S1*)	62 bp (*Y-W*)	193bp (*L2-cox2*)	-
		Apis mellifera ruttneri	MN714162	161	16,577	1,003 bp (*rrnS-E*), 60 bp (*E-S*)	65 bp (*Y-W*)	259 bp (*L-cox2*)	n/a
		Apis mellifera sahariensis	NC035883	162	16,569	1,230 bp (*rrnS-E*), 60 bp (*E-S1*)	65 bp (*Y-W*)	259 bp (*L2-cox2*)	n/a
		Apis mellifera scutellata	KJ601784	163	16,411	876 bp (*rrnS-E*), 60 bp (*E-S*)	59 bp (*Y-W*)	211 bp (*L2-cox2*)	-
		Apis mellifera sinisxinyuan	MN733955	164	16,886	978 bp (*rrnS-E*), 60 bp (*E-S1*); 150 bp (*S1-M*)	53 bp (*Y-W*)	446 bp (*L2-cox2*)	101 bp (*cox3-G*), 106 bp (*R-N*)
		Apis nigrocincta	KY799147	165	>15,855	≥607 bp (*rrnS-S1*), 204 bp (*M-Q*)	-	86 bp (*L2-cox2*)	n/a
		Apis nuluensis	NC036235	166	15,843	570 bp (*rrnS-S1*), 224 bp (*M-Q*)	-	30 bp (*L2-cox2*)	n/a
	Apidae / Apinae / Corbiculata / Bombini	*Bombus asiaticus*	MH998259	167	>19,752	≥3,711 bp (*Q-M*), 122 bp (*M-A*), 103 bp (*I-nad2*)	74 bp (*Y-W*)	67 bp (*cox1-L2*)	226 bp (*S2-nad1*)
		Bombus consobrinus	MF995069	168	17,966	191 bp (*Q-R*), 2,008 bp (*R-A*)	91 bp (*Y-W*), 191 bp (*W-cox1*)	71 bp (*L2-cox2*)	n/a
		Bombus hypocrita sapporensis	NC011923	169	15,468	392 bp (*M-A*), 126 bp (*I-nad2*)	-	72 bp (*L2-cox2*)	n/a
		Bombus ignitus	NC010967	170	16,434	859 bp (*Q-M*), 55 bp (*M-A*), 123 bp (*I-nad2*)	-	79 bp (*L2-cox2*)	143 bp (*F-nad5*), 102 bp (*nad4L-P*), 226 bp (*S2-nad1*)
		Bombus kashmirensis	MH998261	171	>16,793	≥871 bp (*Q-A*), 97 bp (*A-M*), 247 bp (*M-I*), 101 bp (*I-nad2*)	49 bp (*Y-W*)	180 bp (*L2-cox2*)	n/a
		Bombus lapidarius	KT164641	172	>17,817	≥1,534 bp (*Q-A*), 94 bp (*M-I*), 123 bp (*I-nad2*)	74 bp (*Y-W*)	792 bp (*cox1-L2*)	-
		Bombus pyrosoma	MH998260	173	>18,897	≥2,845 bp (*Q-A*), 200 bp (*M-I*), 83 bp (*I-nad2*)	100 bp (*Y-W*)	58 bp (*cox1-L2*), 75 bp (*L2-cox2*)	289 bp (*nad5-H*), 195 bp (*S2-nad1*)
		Bombus sibiricus	MH998258	174	>20,048	≥4,549 bp (*Q-A*), 155 bp (*I-nad2*)	72 bp (*Y-W*)	104 bp (*L2-cox2*)	117 bp (*S2-nad1*)
		Bombus terrestris terrestris	MK570129	175	17,232	(*Q-M-M-A*): 231 bp (*Q-M*), 52 bp (*M-M*), 977 bp (*M-A*), (*A-I-I-nad2*): 83 bp (*A-I*), 32 bp (*I-I*), 96 bp (*I-nad2*)	61 bp (*C-Y*), 16 bp (*Y-W*)	(*cox1-L2-L2-cox2*): 37 bp (*cox1-L2*), 17 bp (*L2-L2*), 68 bp (*L2-cox2*)	(*S1-F-F-nad5*): 34 bp (*F-F*), 88 bp (*F-nad5*), 144 bp (*nad4L-P*), 247 bp (*S-nad1*)
		Bombus terrestris lusitanicus	NC045178	176	17,049	(*Q-M-M-A*): 247 bp (*Q-M*), 54 bp (*M-M*), 886 bp (*M-A*), 81 bp (*A-I*), 196 bp (*I-nad2*)	61 (*C-Y*), 16 bp (*Y-W*)	(*cox1-L2-L2-cox2*): 37 bp (*cox1-L2*), 17 bp (*L2-L2*), 68 bp (*L2-cox2*)	n/a
		Bombus waltoni	NC045283	177	19,349	4,143 bp (*Q-A*)	-	34 bp (*cox1-L2*)	n/a
	Apidae / Apinae / Corbiculata / Meliponini	*Melipona scutellaris*	NC026198	178	14,862	99 bp (*rrnS-I*), 67 bp (*M-nad2*)	61 bp (*Y-cox1*)	-	n/a
	Apidae / Xylocopinae	*Xylocopa appendiculata*	KX494104	179	>14,655	≥121 bp (*rrnS-A*), 88 bp (*A-I*), 97 bp (*I-nad2*)	64 bp (*Y-W*)	-	-
	Apidae / Eucerini	*Eucera floralia*	KX494108	180	>16,038	≥994 bp (*V-A*), 77 bp (*A-I*), 59 bp (*I-M*), 90 bp (*M-Q*), 138 bp (*Q-nad2*)	-	-	-
